# Bioassay-guided isolation and characterization of lead antimicrobial compounds from *Acacia hydaspica* plant extract

**DOI:** 10.1186/s13568-022-01501-y

**Published:** 2022-12-15

**Authors:** Tayyaba Afsar, Suhail Razak, Ali Almajwal, Maria Shabbir, Khushbukhat Khan, Janeen Trembley, Nawaf W. Alruwaili

**Affiliations:** 1 Department of Community Health Sciences, College of Applied Medical Sciences, King Saud University, Riyadh, Kingdom of Saudi Arabia; 2 Department of Healthcare Biotechnology, Atta-ur-Rahman School of Applied Biosciences(ASAB), National University of Sciences and Technology, Islamabad, Pakistan; 3 Minneapolis VA Health Care System Research Service, Minneapolis, MN USA; 4 Department of Laboratory Medicine and Pathology, University of Minnesota, Minneapolis, MN USA; 5 Masonic Cancer Center, University of Minnesota, Minneapolis, MN USA

**Keywords:** Methyl gallate and catechin 3-*O*-gallate, Microbial infections, NMR spectroscopic analysis, Broth dilution assay, Bacterial strains

## Abstract

*Acacia hydaspica* possesses varied pharmacological attributes. We aimed to examine the antimicrobial potential and isolate the active antimicrobial metabolites**.** The plant extract was fractionated and the antimicrobial activity of the crude extract, fractions and compounds was tested by agar well diffusion and agar tube dilution and broth dilution methods. Bacterial strains selected for bioactivity testing were *Staphylococcus aureus*, *Enterococcus faecalis*, *Bacillus subtilis*, *Escherichia coli*, *Pseudomonas aeruginosa*, *Klebsiella pneumoniae*, *Acinetobacter baumannii* while selected strains from kingdom fungi were *Candida albicans*, *Cryptococcus neoformans*, *Fusarium solani* and *Aspergillus*. The active compounds were isolated from *Acacia hydaspica* by bioassay-guided fractionation and identified by nuclear magnetic resonance and spectroscopic techniques. *S. aureus* cell surface proteins, Autolysins (Atl), Clumping factor A (ClfA), and Fibronectin Binding Proteins (FnBP), were molecularly docked with Catechin 3-*O*-gallate (CG) and Methyl gallate (MG) and binding energy and molecular interactions between the proteins and compounds were analyzed. Ethyl acetate (AHE) and Butanol (AHB) fractions of *A. hydaspica* were the most active fractions against tested microbial strains. Therefore, both were subjected to bioassay-directed fractionation which led to the isolation of one pure active antimicrobial AHE and one active pure compound from AHB fraction besides active enriched isolates. Methyl-gallate (MG) and catechin-*3*-gallate (CG) are active compounds extracted from AHE and AHB fractions respectively. In antibacterial testing MG significantly inhibited the growth of *E. coli* (MIC_50_ = 21.5 µg/ml), *B. subtilus* (MIC_50_ = 23 µg/ml) and *S. aureus* (MIC_50_ = 39.1 µg/ml) while moderate to low activity was noticed against other tested bacterial strains. Antifungal testing reveals that MG showed potent antifungal activity against *F. solani* (MIC_50_ = 33.9 µg/ml) and *A. niger* (MIC_50_ = 41.5 µg/ml) while lower antifungal activity was seen in other tested strains. AHB fractions and pure compound (CG) showed specific antibacterial activity against *S. aureus* only (MIC_50_ = 10.1 µg/ml) while compound and enriched fractions showed moderate to no activity against other bacterial and fungal strains respectively. Molecular docking analysis revealed that CG interacted more strongly with the cell surface proteins than MG. Among these proteins, CG made a stronger complex with ClfA (binding affinity − 9.7) with nine hydrophobic interactions and five hydrogen bonds. Methyl gallate (MG) and catechin 3-*O*-gallate (CG) are the major antimicrobial compound from *A. hydaspica* that inhibit the growth of specific microbes. The occurrence of MG and CG endorse the traditional antimicrobial applicability of *A. hydaspica*, and it can be a legitimate alternative to control specific microbial infections.

**Supplementary Information:**

The online version contains supplementary material available at 10.1186/s13568-022-01501-y.

## Introduction

Scientific research on raw or crude herbs containing several chemical constituents has attracted attention towards the isolation and synthesis of pure phytochemicals and the chemical preparation of specific structural analogues. There is no doubt that several of our conventional modern-day medicines either directly or indirectly resulted from natural products. World Health Organization (WHO) declared that traditional medicines are harmless cures for ailments of both microbial and non-microbial origins (WHO 1978). Several plants have been used for the reason that they possess antimicrobial potential owing to the occurrence of phytochemicals (Janssen et al. 1976; Saxena et al. 1994). Albeit drug detection from remedial plants does not always provide new chemical structures yet known compounds with novel biological action give rise to important drug leads. Bioassay-guided fractionation techniques are used to isolate compounds of interested bioactivity from complex mixtures and it involves separation and analytical techniques, where fractionated material is sequentially tested for activity in a bioassay and purity by analytical methods (van Beek et al. 2009). Depending on the number of extracts to be screened either the low-throughput screening (LTS) or high-throughput screening (HTS) methods are operated (Okwu 2001; Valsaraj et al. 1997).

Various members belonging to the *Acacia* genus have also been accounted to be useful in conventional drugs to cure several microbial infections and an array of related diseases including malaria, sore throat (aerial part) and toothache (bark) (Kubmarawa et al. 2007). *A. nilotica* is considered an auspicious resource for antibacterial drugs due to the possession of antimicrobial metabolites. Negi and Dave demonstrated the antimicrobial activity of *A. catechu* methanol extract against six types of pathogenic and non-pathogenic microorganisms viz. *B. subtilis*, *S. aureus*, *S. typhi*, *E. coli*, *P. aeruginosa* and *C. albicans*. *S. aureus* showed maximum susceptibility. Both gram-negative and gram-positive strains were affected by methanol extract. They concluded that the antibacterial and antifungal perspective of leaf extracts of *A. catechu* was attributed to its extraordinary terpene quantity (Negi and Dave 2010). Arias and colleagues reported the antibacterial potency of seven ethanol and three aqueous extracts obtained from various parts (leaves, stem and flowers) of *A. aroma* against 163 strains of multi-resistant bacteria comprising both gram-positive and gram-negative strains via disc diffusion method (Arias et al. 2004). Biological activities should be correlated with phytochemicals to corroborate activity with respective chemical constituents (van Vuuren 2007).

*Acacia hydaspica* R. Parker belongs to the family Leguminosae and is reported to be common in Iran, India and Pakistan (Zargari 1997). It is treated as a synonym of *A. eburnea* (Chakrabarty and Gangopadhyay 1996). The bushy shrub or tree is found in scrub and open rocky, dry areas and sandy soils. The traditional healers of India use various parts of the plant for the treatment of diarrhoea, the leaves are helpful in the cure of dysentery (Arulappan et al. 2015). *A. hydaspica* showed significant antioxidant (Afsar et al. 2016, 2018), anticancer (Razak et al. 2019), anti-inflammatory potentials in our previous investigations (Afsar et al. 2015). The current study aims to scientifically validate the antimicrobial activity of the plant. Furthermore, bioassay-guided isolation of active anti-microbial agents was done to identify the constituents responsible for the activity.

## Methods

### Plant collection

The collection of aerial parts of *A. hydaspica* was carried out from the Kirpa area of Islamabad, Pakistan. The plant was recognized by its local term and authenticated by Dr. Rizwana, Department of Plant Sciences, Quaid-i-Azam University, Islamabad and Dr. Sumaira Sehreen, Associate Curator, Museum of Natural History, Islamabad, Pakistan. A voucher sample of plant-acquired accession No. 0642531 was submitted to the Herbarium of Pakistan Museum of Natural History, Islamabad.

### Preparation of extract

After collection and identification, the plant sample was dried in an aerated but shaded area till the complete removal of moisture. The dried plant was ground by an electrical grinder (60-mesh topology Willy Mill) to obtain a fine powder of plant sample and then this plant material was utilized for solvent extraction. The powder (3 kg) plant sample was soaked in 6 l of crude methanol (95%) solution for 72 h at 25 °C and extracted thrice. Filteration was done using Whatman filtrating paper No. 1 and the filtrate was evaporated at 40 °C using a rotary vacuum evaporator (Panchun Scientific Co. Kaohsiung, Taiwan) and the crude methanol extract (AHM) was obtained.

### Fractionation

Fractional separation of crude methanol extract was done by solvent–solvent extraction. Briefly crude methanol extract (12 g) was dissolved in distilled water (500 ml) in a separating funnel of 1000 ml capacity and successively partitioned with *n-*hexane, chloroform, ethyl acetate and *n-*butanol to obtain their soluble fractions. The filtrate was concentrated using a rotary evaporator. The scheme of fractionation is summarized in our previous publications (Afsar et al. 2016). The crude methanol extract (AHM) and its five subsequent fractions: *n*-hexane (AHH), chloroform (AHC), ethyl acetate (AHE), *n*-butanol (AHB) and remaining water (aqueous) fraction (AHA) were weighed to determine the resultant mass and quantified in terms of percentage of dried plant sample. Fractions were stored at 4 °C until used for experimentation.

### Initial antimicrobial screening of crude extract/fractions

The initial antibacterial and anti-fungal screening of crude extract AHM and its derived fractions (AHE, AHH, AHC, AHB and AHA) were carried out using the agar well diffusion method and agar tube dilution technique respectively (Boyanova et al. 2005; Duraipandiyan and Ignacimuthu 2009).

#### Microorganisms and culture conditions

The antibacterial activity of extract/fractions was checked against 6 bacterial strains, two Gram-positive e.g., *Staphylococcus aureus* (ATCC 6538) and *Micrococcus luteus* (ATCC 10240), and four Gram-negative E.g. *Bacillus subtilis* (ATCC 6633), *Escherichia coli* (ATCC 15224), *Klebsiella* *pneumoniae* (MTCC 618), *Pseudomonas aeroginosa* (ATCC 27853). Bacteria used for inoculum preparation were grown in a nutrient broth medium (MERCK). Antifungal activity of *A. hydaspica* methanol extract/fractions was analyzed against 4 fungal strains *i.e. Aspergillus flavus* (ATCC 0064), *Aspergillus fumigatus* (ATCC 66), *Aspergillus niger* (ATCC 0198), *Fusarium solani* (ATCC 0300). Fungal strains were maintained on Sabouraud Dextrose Agar (MARK) slants. Bacterial culture turbidity was checked using McFarland 0.5 of Mc Farland standard turbidity scale corresponding to about 1.5 × 106 colony forming unit (CFU) per ml The microbial suspensions were standardized by adjusting the optical density to 0.1 for bacteria and 0.09 for yeast at 600 nm (Jenway 6105 UV/Vis spectrophotometer, 50 Hz/60 Hz). These dilutions correspond to about 2.5 × 105 spores/ml for yeast and 10^6^ CFU/ml for bacteria.

### Antibacterial assay

#### Sample preparation

Serial dilutions were made for all crude fractions by using DMSO at a concentration of 32, 16, 8.0, 4.0 and 2.0, 1.0, 0.5, and 0.25 mg/ml.

### Assay procedure

2 g of nutrient agar (NA) was mixed with 100 ml distilled water (pH 7.0) to make a nutrient agar medium and then autoclaved. NA medium was cooled up to 45 °C and poured into pre-labelled Petri plates (14 cm). Then 75 ml of nutrient broth media (NB) was dispensed in each plate and left for solidification. Ten wells (8 mm) per plate were prepared 8 mm by using a sterile borer. Swabbing of each test bacterial strain was done or a bacterial lawn was made on the plates with the help of a sterile cotton swab to make sure equal growth over the entire surface area. 100 μl of each dilution of extract/fractions was added into the respective well, cefixime (1 mg/ml) as a positive control and DMSO was used as a vehicle (negative) control in each plate. For each bacterial strain plates were made in triplicate. After sample loading plates were incubated for 24 h at 37 °C. Next to incubation, the spans of the zone of growth inhibition were measured in millimeters (mm) with the help of a Vernier caliper, and the antibacterial activity was assessed thrice.

### Antifungal assay

#### Preparation of samples

A quantity of 12 mg/ml stock solutions of extract/fractions was made and 67 µl of the sample was used from the initial stock. Terbinafine (12 mg/ml in DMSO) was used as positive control while DMSO was used as a negative control.

#### Assay procedure

Media for fungal growth was prepared by dissolving 6.5 g/100 ml in distilled water (pH 5.6). 4 ml of Sabouraud dextrose agar (SDA) was dispensed into screw-capped test tubes and autoclaved for 20 min at 121 °C. After sterilization, the tubes in the autoclave were allowed to 50 °C. 67 μl of test samples from the initial stock solution was added into 4 ml of non-solidified sterilized SDA media to get 200 μg/ml concentration of extract in the media. After the addition of test samples, the tubes were placed at room temperature at a slanting position for solidification. The tubes were inoculated after solidification with a 4 mm diameter piece of fungus extracted from a 7 days old fungal culture. Positive and negative reference sample tubes were inoculated similarly. Next, the sample tubes were kept at 28 °C for 7 days. After that, the antifungal activity was examined by quantifying the linear fungal growth in mm. The growth impediment in the test sample was calculated by comparing the growth in the vehicle control tube. The percentage of growth obstruction was calculated by the following equation

$$\mathrm{Percentage\, growth\, hindrance}=100-\left[\frac{{\mathrm{Linear\, growth \,in \,plant\, extract}}/{\mathrm{fraction\, treated \,tube}}\, (\mathrm{mm})}{{{{\mathrm{Linear\, growth\, in \,vehicle}}\,(\mathrm{DMSO})}\,{\mathrm{control }\,(\mathrm{mm})\,\mathrm{ tube}}}}\times 100\right]$$

### Bioassay guided fractionation

After the initial antimicrobial screening of crude extract/fractions, AHE and AHB were selected based on their efficacy against microbial strains and lower MIC values for further screening and isolation of bioactive antimicrobial compounds.

### Fractionation of AHE and antimicrobial activity

#### Vacuum liquid chromatography (VLC)

Detail fractionation procedure for ethyl acetate fraction is reported already in our previous investigations (Afsar et al. 2017, 2018). Briefly, 10 g AHE was dissolved in Dichloromethane (DCM) mixed with neutral acid wash (supercell NF) and dried down completely with a rotary evaporator (Buchi Rotavapor 200). Sintered glass column (diameter 5 cm) is used for VLC that is attached to round bottom flak at the bottom to collect samples. 3/4 volume of the column was packed with silica gel (230–400 mesh) as a dry stationary phase and the AHE sample was loaded over the silica layer. The column was eluted with DCM:MeOH solvents as a mobile phase in order of increasing polarity, starting with 100% DCM (Dichloromethane) to 100% MeOH (Methanol) by using a vacuum applied through a vacuum line connected with the column (Afsar et al. 2018; Maurya et al. 2018; Pelletier et al. 1986). Fractions were collected at each polarity step. After VLC separation AHE was fractionated into 12 fractions in the following ratios of DCM: MeOH; 10:0, 9:1, 8:2, 7:3, 6:4, 5:5, 4.5:5.5, 4:6, 3.5:6.5, 3:7, 2:8, 1:9, 0:10 (v/v). Then these 12 fractions (VLC-AHE/F1-F10) were subjected to antimicrobial testing. Micro broth dilution assay was selected for antimicrobial activity testing. The detailed protocol of micro broth dilution assay is described in a later section.

### Antimicrobial screening of isolated fractions using Broth dilution assay

Antimicrobial screening of fractions obtained at each isolation step was performed by broth dilution protocol based on the NCCLS (National Committee for Clinical Laboratory Standards) procedures for antimicrobial vulnerability testing, 14th informational Suppl, NCCLS; M100-S14: 2002, Wayne, PA (Wayne 2002). The antimicrobial activity isolates were checked against 7 bacterial, and 4 fungal strains. Bacterial strains selected for bioactivity testing were *S. aureus* (ATCC 43300), *E. faecalis* (ATCC 51299), *B. subtilis* (ATCC 6633), *E. coli* (ATCC 25922), *P. aeruginosa* (ATCC 27853), *K. pneumoniae* (ATCC 13883), *A. baumannii* (ATCC 19606) while selected strains from kingdom fungi were *C. albicans* (ATCC 10231), *C. neoformans* (*ATCC* 66031), *F. solani* (ATCC 0300) and *A. niger* (ATCC 0198).

#### Sample preparation

The sample was dried completely and 10 mg/ml stock of each isolate was prepared in DMSO. Serial dilutions were prepared from this stock to get the MIC values of active isolates. MIC is the lowest concentration (expressed as mg/l or μg/μl) of an antimicrobial agent that inhibits the growth of microorganisms (showing no turbidity). Initial screening of antimicrobial activity was performed using 200 µg/ml concentration of each fraction, serial dilutions were made for determining the MIC of active isolates.

#### Procedure

Bacteria and fungi were grown overnight in 15 ml tubes with shaking (225RPM) at the optimal growth temperature for each organism. The turbidity of microbial cultures was adjusted by adding appropriate culture media depending on the test organism under testing, to obtain turbidity equivalent to the 0.5 McFarland test scale using a spectrophotometer at 600 nm OD (Balouiri et al. 2016; Wikler 2006). The diluted culture media was added to the 96-well microtiter plates (195.0 µl/well). The test samples were made in DMSO and pipetted to the wells at a concentration of 2.5% of the good volume (5.0 µl of the sample per well). The plates were wrapped using parafilm to prevent evaporation and incubated overnight (12–18 h) at the test organism’s optimal growth temperature (*Cryptococcus neoformans* requires a longer incubation typically 24–48 h). The plates were analyzed by recording the optical density (OD) at 600 nm. Data were analyzed as follows:

$$\mathrm{Percentage\, growth \,inhibition}\,(\mathrm{\% GI})=\frac{\mathrm{Abs \,of\, bacterial \,control}-\mathrm{Abs\, of\,the\, test \,sample}}{\mathrm{Abs\, of \,bacterial \,control }}\times 100$$

Blank subtraction was done by subtracting the absorbance (Abs) value of media blanks from each Abs before calculating the % GI.

VLC–AHE fractions with potent antimicrobial activity were mixed and subjected to flash chromatography for further bioactivity-targeted purification of active compounds.

### Flash liquid chromatography (FC)

Flash chromatography was done on Teledyne ISCO Combi-flash RF-200 automated system. VLC-AHE/F4-F6 fractions (4 g) were combined using methanol and mixed in neutral acid wash standard super cell NF and dried down completely by a rotary evaporator. The dried sample was loaded on Redisep Column (RediSep^®^ Normal-phase Silica Flash Column, 69-2203-340, TELEDYNE ISCO). The spectrum was monitored at all wavelengths (200–780 nm) with a total run time of 89.2 min. The pattern of step gradient was obtained by holding the mobile phase to the same gradient of the solvent mixture at which the peak starts to appear until the whole peak becomes eluted. 146 fractions collected with FC were pooled into 12 fractions based on their similar TLC (95:5 methanol:chloroform) pattern (observed under UV detection at 254 and 365 nm and staining with Vanillin-HCL reagent) and chromatogram spectral peak appearance. These 12 fractions were submitted for bioactivity testing. Out of these 12 fractions (ISCO-AHE/F1-F12), the fractions which showed promising antimicrobial potential against various tested strains were selected. The MIC_50_ of the active fraction was calculated and the sample was subjected to NMR and mass spectroscopic analysis studies for identification of metabolite. Analytical HPLC indicated the purity of the compound.

### Bioassay-guided isolation of lead compounds from AHB fraction

#### Vacuum liquid chromatography

15 g of AHB was fractionated by VLC over reverse phase C18 (LiChroprep C18) using gradient elution with polar to nonpolar solvent (dH_2_O:MeOH), starting with 100% dH_2_O and ends with 100% DCM. Fractions were collected at each polarity gradient to yield 10 fractions (VLC-AHB/F1-F10). Fractions were dried using a rotary evaporator. Similar to AHE fractions, these fractions were tested for antimicrobial activity, and the active fraction VLC-AHB/F1 was afterwards refined by Sephadex LH 20-column chromatography.

#### Sephadex LH 20-column chromatography

The VLC-AHB/F1 (1.5 g) was chromatographed on Sephadex LH-20 (3 cm × 120 cm) using MeOH as eluent. The volume of each fraction collected was set to 500 ml and 20 fractions were collected. Fractions were then combined based on a similar TLC (95:5 methanol:chloroform) pattern (observed underneath UV light of 254 nm and 365 nm wavelengths, or staining with Vanillin-HCL reagent) and got eight fractions (Sp-AHB/F1-F8). These eight fractions were submitted for bioactivity screening and fraction “Sp-AHB/F4” was shown to be the most potent antimicrobial fraction. ^1^H NMR and HPLC analysis indicated that Sp-AHB/F4 needed further purification. Sp-AHB/F4 fraction was further purified by semi-prep RP- HPLC.

#### Semi-preparative reverse phase high-performance liquid chromatography

Semi-prep RP-HPLC was conducted on Agilent HPLC 1260 series using Grace Vision HT C18 column (10 μm; 10 × 250 mm, USA). The fractions were eluted using mobile phase A: H_2_O (purified by a Milli-Q Water Purification system (Millipore, MA, USA) and mobile phase B: acetonitrile. Method: 0–5 min; 15% B in 85% A (isocratic run), 5–25 min; up to 70% B in 30% A, 25–27 min; up to 100% B, 27.1–32 min; 15% B in 85% A (isocratic run). The flow rate was 3 ml/min and the injection volume was 50 μl. The spectrum was monitored at λ 220, 254, 280 and 330 nm. The Fractions were pooled based on chromatogram peaks into 10 fractions (RP-HPLC-AHB/F1-F10) eluted at different retention times with different patterns of UV absorption. These 10 fractions collected by Semi-prep RP-HPLC were tested again for antibacterial activities. RP-HPLC-AHB/F5 showed the most promising antimicrobial potential. Analytical HPLC indicated the purity of the compound. NMR and mass spectroscopic techniques were utilized to identify the compound structure.

### HPLC–DAD screening

A chromatography experiment was run to validate the purity of compounds by employing HPLC–DAD equipped with a Grace Vision HT C18 column (1.8 µm 2.1 × 50 mm column, USA). Stock solutions of compounds were made in methanol at 0.5 mg/ml of concentration. Briefly, mobile phase A was H_2_O (Cleansed by a Milli-Q Water Purification system (Millipore, MA, USA) and mobile phase B was acetonitrile. A gradient of time 0–5 min (isocratic run) for 85% A in 15% B, 50–25 min for 15 to 100% B, followed by an isocratic 100% B till 30 min of the run. The flow rate was 1 ml/min and the injection volume was 20 µl. All the test mixtures were scrutinized at 220, 254, 280, 330, and 360 nm wavelengths. Each time column was revamped for 10 min before the subsequent run. The whole chromatographic tasks were accomplished at ambient temperature.

#### Nuclear magnetic resonance spectroscopy (NMR)

NMR Spectrum (^1^H and ^13^C-NMR) for each compound was acquired on a Varian 600 MHz CDD NMR machine with 1H and 13C frequencies of 599.664 and 150.785 MHz, respectively at 25 °C. Spectra of active compounds were obtained in Methanol-d4 and DMSO-d6. Broad-spectrum 1D and 2D Fourier transform methods were exploited as crucial to achieve unambiguous signal assignments. Additionally, 2D shift-correlation analysis (H–H COSY with long-range connectivity; C–H correlation via ^*J*^CH), ^1^H-coupled ^13^C spectra and selective ^1^H-decoupling were employed extensively to determine long-range ^*J*^CH coupling constants and allocate all quaternary carbons unequivocally (DEPT). The DEPT study of resolution improved spectra (Peak picking, integration, and multiplet analysis) was executed on Varian NMR and ACD/NMR processor (Academic Edition software). ^1^H and ^13^C chemical shifts were described in ppm comparative to DMSO-d6 (*δ* 2.5 and *δ* 39.5 for ^1^H and ^13^C respectively), CD3OD (*δ* 3.31, 4.78 for ^1^H and *δ* 49.2 for ^13^C) or inner Me_4_Si standard (TMS, *δ* = 0.0). The spectral information of isolated compounds is coordinated with published data.

#### Mass spectroscopy

The minute amount of the compound sample was dissolved in methanol for MS analysis. Low-resolution ESI–MS of isolated compounds were obtained utilizing liquid chromatography–mass spectrometry (LC–MS) (Agilent system).

#### Molecular docking

As both CG and MG showed specifically significant antimicrobial activity against *S. aureus* compared to other strains so we have selected this bacterial specie for docking studies. Several cell surface proteins are present in *S. aureus,* among which three proteins: Autolysins (Atl), Clumping factor |A (ClfA), and Fibronectin binding protein (FnBP) play important roles in its virulence by promoting its division, colonization, and surface adhesion, respectively (Foster 2019). The complete tertiary structures of *S. aureus* bacterial cell surface protein Atl (ID: AF-Q6GI31), ClfA (ID: AF-Q53653), and FnBP (ID: AF-A0A0H2XKG3) were obtained from AlphaFold database (David et al. 2022) after ensuring these protein structures unavailability in RCSB Protein Data Bank (Burley et al. 2021). Chemical structures for Catechin 3-*O*-gallate and Methyl gallate were retrieved from the database PubChem (Kim et al. 2019). Their chemical IDs are CID: 5276454 and CID: 7428, respectively. The online server tool CB dock was used to perform rigid docking between bacterial cell surface protein and molecular compounds (Liu et al. 2020). The best-docked complex was selected based on the lowest vina score and largest cavity size.

Statistics: Values are expressed as Mean ± SEM (n = 3). Data were analyzed by One-way ANOVA followed by Tukey’s comparison test using Graph pad prism 9 software.

## Results

The aerial parts (bark, twigs, and leaves) of *A. hydaspica* were collected and subjected to bioassay-guided extraction and fractionation as described in the “Methods” section. Graphic summarization of the process is shown in Additional file 1: Figure S1.

### Antibacterial activity of crude extract/fractions

Crude methanol extract of *A. hydaspica* and its consequent fractions were checked out for antibacterial potency using Gram + ive and Gram −ive bacteria. The antibacterial activity of extract/fractions is calculated by measuring the zone of inhibition in mm and minimum inhibitory concentration (MIC) in mg/ml (Table 1). The activity shown by plant samples is compared with standard antibiotics (Cefixime). The result indicated that AHE possesses strong antibacterial activity and maximum zone of inhibition (28 ± 1.0 mm) against *E. coli*, followed by AHM (11 ± 0.5 mm), while the rest of the fractions did not inhibit the growth of *E. coli.* Similarly, maximum inhibitory activity was exhibited by AHE against *B. subtilus* and *S. aureus.* AHE showed 25 ± 0.5 mm and 23 ± 1.0 mm zone of inhibition against *B. subtilus* and *S. aureus* respectively, followed by AHB (20 ± 0.5 mm against *B. subtilus* and 22 ± 1.5 against *S. aureus*). AHM showed moderate activity against *B. subtilus* (18 ± 1.5 mm) and the least (10 ± 0.5 mm) inhibitory activity against *S. aureus. Bacillus subtilus* and *S. aureus* strains showed resistance to AHH, AHC and AHA fractions as indicated by no zone of inhibition. *Bordetella bronchiseptica* and *M. luteus* showed resistance to all of the tested extract/fractions except AHE, which showed a moderate (20 ± 1.5 mm) effect against *B. bronchiseptica* and a low (8 ± 1.0 mm) effect against *M. luteus*. *P. aeroginosa* strain showed susceptibility from non to high. AHC showed no effect, while AHH, AHB and AHA showed low (7 ± 0.5 mm, 9 ± 1.5 mm and 8 ± 0. 5 mm, respectively), AHM showed moderate (19 ± 0.5 mm) and AHE showed slightly high (17 ± 0.5 mm) zone of inhibition. AHM (10 ± 1.0 mm) showed the least antimicrobial activity and AHE exhibited moderate (16 ± 0.5 mm) against *K. pneumoniae* while the remaining fractions showed no activity. MIC values recorded for *A. hydaspica* extract/fractions ranged from 0.5 to 8.0 mg/ml. The lowest MIC was recorded for AHE against *B. subtilus, S. aureus* (Gram +ive bacteria) and *E. coli* (Gram −ive bacteria), besides AHB also showed the lowest MIC against *S. aureus.*

**Table 1:** Antibacterial activity of *A. hydaspica* methanol extract and its derived fractions at 30 mg/ml concentration

Samples	Zone of inhibition (mm)						
	*E. coli*	*B. subtilus*	*S. aureus*	*B. bronchiseptica*	*M. luteus*	*P. aeroginosa*	*K. pneumoniae*
AHM	11 ± 0.5 (MIC = 4.0 mg/ml)	18 ± 1.5 (MIC = 4.57 mg/ml)	10 ± 0.5 (MIC = 5.0 mg/ml)	0	0	19 ± 0.5 (MIC = 5.5 mg/ml)	10 ± 1.0 (MIC = 5.0 mg/ml)
AHH	0	0	0	0	0	7 ± 0.5 (MIC = 15.0 mg/ml)	0
AHC	0	0	0	0	0	0	0
AHE	28 ± 1.0 (MIC = 0.5 mg/ml)	25 ± 0.5 (MIC = 0.5 mg/ml)	23 ± 1.0 (MIC = 0.5 mg/ml)	20 ± 1.5 (MIC = 1.0 mg/ml)	8 ± 1.0 (MIC = 8.0 mg/ml)	17 ± 0.5 (MIC = 3.5 mg/ml)	16 ± 0.5 (MIC = 4.0 mg/ml)
AHB	0	20 ± 0.5 (MIC = 1.0 mg/ml)	23 ± 1.5 (MIC = 0.5 mg/ml)	0	0	9 ± 1.5	0
AHA	0	0	0	0	0	7 ± 0. 5 (MIC = 15.0 mg/ml)	0
Cefixime	38 ± 0.5	30 ± 1.5	34 ± 0.5	35 ± 1.0	32 ± 1.0	42 ± 0.5	30 ± 1.5
DMSO	0	0	0	0	0	0	0

Minimum inhibitory concentrations are presented as mg/ml

Values are expressed as mean ± SEM. 8–13, low; 14–19, moderate; 20-above high

AHM: *A.* *hydaspica* methanol extract; AHH: *A. hydaspica* n-hexane fraction; AHE: *A. hydaspica* ethyl-acetate fraction; AHC: *A. hydaspica* chloroform fraction; AHB: *A. hydaspica* n-butanol fraction; AHA: *A. hydaspica* remaining aqueous fractions

### Antifungal activity

In vitro antifungal potency of *A. hydaspica,* crude extract and its consequent fractions were executed against four different fungal strains. The activity was calculated as percentage growth inhibition relative to the control group. Table 2 illustrates the antifungal potential of *A. hydaspica.* AHM and AHE were shown to be active against all tested fungal strains with variable potency. AHM showed good inhibitory activity against *F. solani* (65 ± 1.5%) and *A. flavus* (70 ± 1.0%) while moderate antifungal activity against *A. niger* (55 ± 2.0%) and *A. fumigatus* (50 ± 0.5%). Significant growth inhibition was recorded in *F. solani* (85 ± 1.5%) and *A. niger* (75 ± 2.0%) strains when treated with AHE, while *A. flavus* (60 ± 0.5%) and *A. fumigatus* (55 ± 1.0%) showed moderate growth inhibition upon treatment with AHE. Treatment with AHB results in moderate growth inhibition of *F. solani* (60 ± 1.5%) while non-significant growth inhibition was noticed against other fungal strains. AHH, AHC and AHA exhibited slight antifungal activity against all tested strains.

**Table 2:** Antifungal activity of *A. hydaspica* methanol extract and its derived fractions

	Percent growth inhibition			
Samples	*F. solani*	*A. flavus*	*A. niger*	*A. fumigatus*
AHM	65 ± 1.5	70 ± 1.0	55 ± 2.0	50 ± 0.5
AHH	15 ± 1.0	20 ± 0.5	10 ± 0.5	15 ± 1.5
AHC	8 ± 0.5	12 ± 1.0	5 ± 0.5	10 ± 1.5
AHE*	85 ± 1.5	60 ± 0.5	75 ± 2.0	55 ± 1.0
AHB*	60 ± 1.5	25 ± 0.5	35 ± 0.5	20 ± 0.5
AHA	10 ± 0.5	5 ± 1.5	7 ± 0.5	15 ± 1.5
Terbinafine	97 ± 1.0	90 ± 2.0	100	100
DMSO	0	0	0	0

Values are expressed as mean ± SEM (*n* = 3). Significant activity = more than 70% growth inhibition; Good activity = 60–70% growth inhibition; Moderate activity = 50–60% growth inhibition; non-significant below 50% growth inhibition

AHM: *A. hydaspica* methanol extract, AHH: *A. hydaspica n-*hexane fraction, AHE: *A. hydaspica* ethyl-acetate fraction, AHC: *A. hydaspica* chloroform fraction, AHB: *A. hydaspica n-*butanol fraction, AHA: *A. hydaspica* remaining aqueous fraction

### Bioassay-guided isolation of antimicrobial fractions

Initial antimicrobial screening led to the selection of the ethyl-acetate (AHE) and *n*-butanol (AHB) fractions for further fractionation of active constituents. The isolated pure active compounds/compound with antimicrobial activity were further tested against respective microbial strains to get MIC_50_ values_._

### Fractionation of AHE and bioactivity testing

Fractionation of AHE was done by VLC and eluted fractions were combined into 12 fractions (VLC-AHE/F1-F12) based on TLC. Antimicrobial activity was checked for all VLC fractions (VLC-AHE/F1-F12) against seven bacterial, two yeast and two fungal strains using a micro-broth dilution assay. The initial antimicrobial screening was performed with a 200 µg/ml dose of eluted fractions. MIC was calculated only for active fractions. VLC-AHE/F4-F6 eluted with a 7:3 to 5:5 ratio of DCM:MeOH solvent showed comparatively high antimicrobial activity against tested strains in comparison to other fractions. Table 3 showed the percentage of antibacterial and antifungal growth inhibition by VLC-AHE/F4-F6. Additional file 2: Tables S1 and S2 indicated the antimicrobial data of all VLC fractions.

**Table 3:** Antibacterial and antifungal activity of VLC-AHE fractions (% growth inhibition)

Antibacterial activity							
Fractions	*S. aureus*	*E. coli*	*P. aeruginosa*	*E. faecalis*	*K. pneumoniae*	*A. baumannii*	*B. subtilis*
AHE/F4	46 ± 0.55	54 ± 0.48*	22 ± 0.23	13.2 ± 0.12	10 ± 0.11	7 ± 0.15	70 ± 0.87*
AHE/F5	43 ± 0.47	58 ± 0.53*	27.1 ± 0.20	11.7 ± 0.23	8 ± 0.27	9 ± 0.20	65 ± 0.76*
AHE/F6	40 ± 0.51	55 ± 0.61*	25.5 ± 0.41	15.2 ± 0.16	9.5 ± 0.11	8 ± 0.27	69 ± 0.77*
Tetracyclin	94 ± 1.01	95 ± 0.99	95 ± 1.03	–	93 ± 0.89	95 ± 1.15	96 ± 1.08
Penicillin G	–	–	–	94 ± 0.87	–	–	–

Antifungal activity				
Fractions	*C. neoformans*	*C. albicans*	*F. solani*	*A. niger*
AHE/F4	11.29 ± 0.56	34.7 ± 0.73	75.6 ± 0.89*	63.2 ± 1.01
AHE/F5	10.92 ± 0.43	32.7 ± 0.59	73.6 ± 0.91*	71.7 ± 0.96*
AHE/F6	9.35 ± 0.28	31.6 ± 0.46	75.5 ± 0.69*	65.2 ± 0.79
Amphotericin B	94.9 ± 1.05	82.70 ± 0.93	90.1 ± 0.92	92.4 ± 1.01

Values are expressed as mean ± SEM (*n* = 3). Significant activity = more than 70% growth inhibition; Good activity = 60–70% growth inhibition; Moderate activity = 50–60% growth inhibition; non-significant below 50% growth inhibition. *Indicate highest growth inhibitory potential (GI %) of fraction, and fractions with * activity were mixed and subjected for further purification and isolation of active metabolite bioactive against specific microbial strain

The VLC-AHE/F4-F6 fractions (4 g) were combined and loaded on the Combi*Flash system* using DCM as solvent: A and MeOH as solvent: B. For the initial 5 min an initial isocratic run with 0% A was performed, then 5.3% B for the next 3.2 min, 7.4% B from 10–28 min and 10.3% B from 29–44 min. Then a linear gradient was selected for the run on Combi*Flash* systems as it provided adequate resolution for most compounds. This gradient was useful for purifying natural products where the desired compound was not known. Additional file 2: Figure S2 indicated the spectral chromatogram obtained from Combi*Flash* systems. 146 fractions collected fractions were pooled into 12 fractions (ISCO-AHE/F1-F12) according to their similar TLC pattern and spectral peaks.

### Antimicrobial screening of ISCO fractions

The results of the antimicrobial activity of isolated ISCO fractions are summarized in Table 4. Initial antimicrobial testing was performed with a 100 µg/ml dose. Results indicated that only one ISCO fraction (ISCO-AHE/F3) showed comparatively high antibacterial and antifungal activity against tested strains in comparison to other fractions, therefore the MIC_50_ was calculated for that fraction only (Table 5). Percentages of bacterial and fungal growth inhibition revealed by each fraction/compound are shown in Additional file 2: Tables S3 and S4. ISCO-AHE/F3 showed maximum growth-inhibiting activity against one Gram −ive (*E. coli*) and two Gram +ive (*B. subtilus* and *S. aureus*) bacteria. Table 5 indicated that the MIC_50_ values of ISCO-AHE/F3 against susceptible strains ranged from 21.5–39.0 µg/ml. ISCO-AHE/F5 is moderately active against *E. coli* (MIC = 151.2 µg/ml) and ISCO-AHE/F7 showed good antibacterial activity only against *B. subtilus* (MIC = 59.3 µg/ml).

**Table 4:** Antibacterial activity of *A. hydaspica*: isolated fractions/compounds from AHE by flash chromatography (ISCO)

Antibacterial activity				Microorganism			
Samples	*Gram *+ *ive*			*Gram −* *ive*			
	*S. aureus*	*E. faecalis*	*B. Subtilis*	*E. coli*	*P. aeruginosa*	*K. pneumoniae*	*A. baumannii*
	% GI	% GI	% GI	% GI	% GI	% GI	% GI
AHE/F3	91 ± 1.17*^,+++^	44 ± 1.23	90 ± 1.71^*,+++^	99 ± 0.99*^,+++^	46 ± 0.87	31 ± 0.98	25 ± 0.56
AHE/F5	21 ± 0.67	18 ± 0.31	46 ± 1.01	62 ± 1.10^+++^	15 ± 0.16	8.0 ± 0.62	11 ± 0.13
AHE/F7	24 ± 0.52	16 ± 0.27	70 ± 1.30^+++^	24 ± 0.18	1.0 ± 0.02	6.6 ± 0.25	− 3 ± 0.05
Tetracycline	95 ± 1.21	–	99 ± 1.79	96 ± 1.43	94 ± 1.13	95 ± 1.11	96 ± 1.17
Penicillin G	–	94 ± 1.66	–	–	–	–	–

Antifungal activity			Microorganism	
	Yeast		Fungi	
Sample	*C. neoformans*	*C. albicans*	*F. solani*	*A. niger*
	% GI	% GI	% GI	% GI
AHE/F3	20.62 ± 0.34	30.3 ± 0.41	87.2 ± 0.94*	86.3 ± 1.0*
Amphotericin B	94.90 ± 1.05	82.70 ± 0.96	90.1 ± 1.01	92.4 ± 1.11

Values are expressed as mean ± SEM (*n* = 3). Significant activity = more than 70% growth inhibition; Good activity = 60–70% growth inhibition; Moderate activity = 50–60% growth inhibition; non-significant below 50% growth inhibition. Symbol (–) represents not done. GI (growth inhibition at 100 µg/ml)

*Indicate highest growth inhibitory potential (GI %) of fraction, and fraction with * activity was subjected for further testing to get MIC_50_ values and structure elucidation

^+++^Indicated significant (p < 0.0001) inhibitory potential of selected fraction compared to other fractions. Data analyzed by One-way ANOVA followed by Tukey’s comparison analysis

**Table 5:** MIC_50_ of AHE/F3 (Methyl gallate) against susceptible microbial strains

Bacterial strains	*S. aureus*	*B. subtilis*	*E. coli*
	MIC_50_	MIC_50_	MIC_50_
	39.1 ± 0.21	23 ± 0.18^+++^	21.5 ± 0.25^+++^

Fungal strains	*F. solani*	*A. niger*
	MIC_50_	MIC_50_
	33.9 ± 0.20	41.5 ± 0.35

Values are expressed as mean ± SEM (*n* = 3). Significant activity = more than 70% growth inhibition; Good activity = 60–70% growth inhibition; Moderate activity = 50–60% growth inhibition; non-significant below 50% growth inhibition. Symbol (−) represents not done, MIC_50_ (Minimum inhibitory concentration resulting in 50% growth inhibition in µg/ml)

^+++^Indicated significant (p < 0.0001) difference of antibacterial activity of MG against *B. subtilis* and *E. coli* in comparison to *S. aureus.* Data were analyzed by one-way ANOVA followed by Tukey’s multiple comparison test

### Bioassay guided fractionation and isolation of compounds from AHB fraction

#### Vacuum liquid chromatography

By using C18 on vacuum liquid chromatographic (VLC) separation column using mixtures of dH_2_O:MeOH in varying proportions. AHB is initially partitioned into 10 fractions (VLC-AHB/F1-F10) based on similar TLC patterns. The antimicrobial activity of VLC fractions (VLC-AHB/F1-F10) was evaluated at 200 µg/ml dose. Results indicate that only VLC-AHB/F1 showed good (75%) growth inhibitory activity against *S. aureus*, while other microbial strains showed resistance. Therefore, the active fraction VLC-AHB/F1 was further purified.

#### Sephadex LH 20-column chromatography

The active fraction VLC-AHB/F1 was further purified on Sephadex LH 20 chromatography using methanol solvents as a mobile phase. Eluted fractions were pooled into 8 fractions (Sp-AHB/F1-F8) based on similar TLC behavior. Eight fractions obtained from Sephadex separation (Sp-AHB/F1-F8) were tested only against *S. aureus* to get the active component. 100 µg/ml dose of each fraction was used for initial screening against *S. aureus*. Results showed that 4 fractions (Sp-AHB/F3-F6) possess antibacterial activity against *S. aureus*. Sp-AHB/F4 showed the lowest MIC_50_ (concentration inhibiting 50% growth of bacteria) of 17.1 µg/ml (Table 6). The remaining 4 fractions (Sp-AHB/F, F2, F7 and F8) showed no activity against S*. aureus* (Additional file 2: Table S5). Therefore, Sp-AHB/F4 fraction was selected for further evaluation.

**Table 6:** Antibacterial activity of Sephadex LH20 and Semi-prep RP-HPLC fractions against *S. aureus*

Samples	Sephadex LH20	
	MIC_50 (µg/ml)_	% GI
AHB/LH20-F3	53.1 ± 0.41	78.3 ± 0.65
AHB/LH20-F4	17.1 ± 0.20^+++^	90.0 ± 0.87*
AHB/LH20-F5	55.2 ± 0.58	76.6 ± 0.81
AHB/LH20-F6	64.9 ± 0.61	70.2 ± 0.92

Samples	Semi-prep RP-HPLC	
	MIC_50 (µg/ml)_	% GI
AHB/RP-HPLC-F5 (CG)	10.1 ± 0.11	92.0 ± 0.85*
Tetracyline	–	96.0 ± 1.01

Values are expressed as mean ± SEM (*n* = 3). Symbol (–) indicated not done, MIC_50_ denoted the minimum inhibitory concentration resulting in 50% growth inhibition in µg/ml and GI represents growth inhibition at 100 µg/ml. *Indicate highest growth inhibitory potential (GI %) of fraction, and fraction with * activity was subjected for further testing to get MIC_50_ values and structure elucidation. ^+++^Indicated significant (p < 0.0001) inhibitory potential of selected fraction compared to other fractions. Data were analyzed by one-way ANOVA followed by Tukey’s multiple comparison test

#### HPLC analysis of Sp-AHB/F4

HPLC examination was performed to check the purity of the Sp-AHB/F4 fraction. HPLC chromatogram of Sp-AHB/F4 showed that it is an enriched fraction that needed further bioassay-guided purification (Additional file 1: Figures S3 and S4). Therefore, Sp-AHB/F4 was further purified by semi-prep HPLC and eluted fractions were submitted for bioactivity analysis.

### Semi-preparative RP-HPLC

Sp-AHB/F4 was further refined by semi-preparative high-performance liquid chromatography (Semi-prep RP-HPLC) using the C18 column. The fractions were eluted using mobile phase A: H_2_O and mobile phase B: acetonitrile. Analytical HPLC by using altered mobile phases was performed for optimization of conditions for fractionation. The spectrum was monitored at λ 220, 254, 280 and 330 nm. The fractions were pooled based on chromatogram peaks in to 10 fractions (RP-HPLC-AHB/F1-F10) eluted at different retention times with different patterns of UV absorption. The Additional file 1: Figure S5 shows the collection pattern of the Semi-prep HPLC chromatograph of Sp-AHB/F4. These 10 fractions (RP-HPLC-AHB/F1-F10) collected by Semi-prep RP-HPLC were tested for antibacterial activity against *S aureus.*

### Antibacterial screening of semi-prep RP-HPLC-AHB fractions

The antibacterial activity of RP-HPLC-AHB/F1-F10 against *S. aureus* was carried out using a micro-broth dilution assay. 100 µg/ml dose of each fraction was used for initial screening against *S. aureus* (Additional file 2: Table S6). Results showed that only one fraction (RP-HPLC-AHB/F5) possesses antibacterial activity against *S. aureus*. RP-HPLC-AHB/F5 was eluted between RT 13.45–14.042 min and showed a wide clear spectral peak at 254 and 280 nm. MIC_50_ (concentration inhibiting 50% growth of bacteria) value of RP-HPLC-AHB/F5 was shown to be 10.1 µg/ml (Table 6). The remaining fractions showed no activity against S*. aureus* therefore RP-HPLC-AHB/F5 was the major component responsible for the antibacterial activity. This fraction was analyzed by spectroscopic and NMR analysis to determine the purity and identity of the compound.

### Structure characterization of purified antimicrobial compounds

#### Identification of antimicrobial compound from AHE

Bioassay-directed fractionation of *A. hydaspica* led to the isolation of one active pure antimicrobial compound from AHE beside the active enriched fractions. The HPLC, ^1^H NMR, ^13^C NMR and MS indicated that the bioactive fraction ISCO-AHE/F3 is a pure compound and the compound is identified as methyl gallate. The detailed ^1^H NMR and ^13^C NMR data are included in Additional file 2: Tables S7 and S8.

#### Methyl gallate (MG)

White needle crystals. (C_8_H_8_O_5_). ESI–MS (−) m/z 183.0534 [M−H].

^1^H NMR (acetone-D6, 600 MHz), δ3.79 (3H, s, OCH3), δ 7.11 (2H, s, H-2, H-6);

^13^C NMR (Acetone-D6, 150.80 MHz) δ 51.0 (OCH3), δ 108.90 (C-2, C-6), δ 120.91 (C-1), δ 137.76 (C-4), δ 145.12 (C-3, C5), δ 166.27 (C=O).

The molecular formula was determined from the MS and ^13^C NMR. 8 Carbons and 5 protons attached to carbon were observed in the ^13^C NMR and ^1^H NMR spectra. To determine the position and number of hydroxyl groups, the NMR solvent was shifted to DMSO-d6 as hydroxyl was not seen with Acetone-d6. ^1^H NMR (DMSO-d6, 600 MHz) clearly reveal the presence two hydroxyls at δ9.44 and one hydroxyl at δ9.11. Close examination of the ^1^H NMR and ^13^C NMR spectrum showed a symmetrical molecule with two aromatic protons, δ 7.11 (2H, s, H-2, H-6), three hydroxyls, two hydroxyls at δ C 145.12 (C-3, C-5), and one hydroxyl at δ C 137.76 (C-4), a methyl δ3.79 (3H, s, OCH3) and an ester carbonyl δ 166.27 (C=O). ^1^H NMR and ^13^C NMR spectra are included in Additional file 1: Figures S6 and S7. The data is consistent with NMR data that have been reported in the literature (Kane et al. 1988). The structure of the compound is revealed to be methyl 3,4,5-trihydroxybenzoate or methyl gallate. The molecular formula of MG was resolute as C_8_H_8_O_5_ showed a molecular peak at m/z 183.0534 [M−H] in its negative–ion ESI–MS spectrum.

#### Identification of antimicrobial compound from AHB

Bioassay-directed fractionation of *A. hydaspica* led to the isolation of one active pure antimicrobial compound from AHB beside the active enriched fractions. The HPLC, ^1^H NMR, ^13^ C NMR and MS indicated that the bioactive fraction RP-HPLC-AHB/F5 is a pure compound and the compound was identified as Catechin 3-*O*-gallate. The detailed ^1^H NMR and ^13^C NMR data are included in Additional file 2: Tables S7 and S8.

### Catechin 3-*O*-gallate (CG)

Golden brown powder, (H_2_O) C_22_ H _18_ O_10_. ESI–MS (−) m/z 441.0988 [M−H].

^1^H NMR (methanol-d4, 600 MHz): δ 7.045 (S, galloyl), δ 5.86 (H-6, J = 2.0 Hz), δ 5.95 (H-8, *d*, J = 2.0 Hz), δ 4.56 (H-2, *d*, J = 7.6 Hz), δ 3.95 (H-3, *m*), δ 2.84 (H-4 α *dd*, J = 16.4 Hz, J = 5.3 Hz), δ 2.5 (H-4 β, *dd*, J = 15.8, 8.2 Hz).

^13^C NMR (Methanol-d4-150.79 MHz): δ 27.07 (*t*, C-4), δ 66.96 (*d*, C-3), δ 81.53 (*d*, C-2), δ 100.66, δ 105.57 (each *d,* C-6 and C-8), δ 105. 57 (s, C-4a), δ 109.18 (galloyl C-2), δ 113.77, δ 114.84 (each *d*, C-2′ and C-5′), δ 119.201 (s, galloyl C-1), δ 130.48 (s, C-1'), δ 139.07 (s, galloyl, C-4), δ 145.24 (s, galloyl, C3), δ 150.34 (s, C-7), δ 155.38, δ 156.16 (each s, C-5 and C-8a), δ165.70 (s, COO-).

The ^1^H NMR and ^13^C NMR of the compound were similar to the assignment of catechin 3-*O*-gallate signals of those reported in previous literature (Davis et al. 1996; Shen et al. 1993). To determine the position of the galloyl group NOESY spectroscopy was performed, which indicates the existence of galloyl ester moiety on C-3. This knowledge, along with the HMBC connectivity, allowed a complete ^13^C assignment to be made and all the protons to be assigned. Consequently, the structure of the compound is concluded to be catechin 3-*O*-gallate and the molecular formula of CG was resolved as C_22_H_18_O_10_ by the ESI–MS (−) signal at m/z 442.088 [M−H]. ^1^H NMR and ^13^C NMR spectra are included in Additional file 1: Figures S6 and S7.

**Fig. 1:**
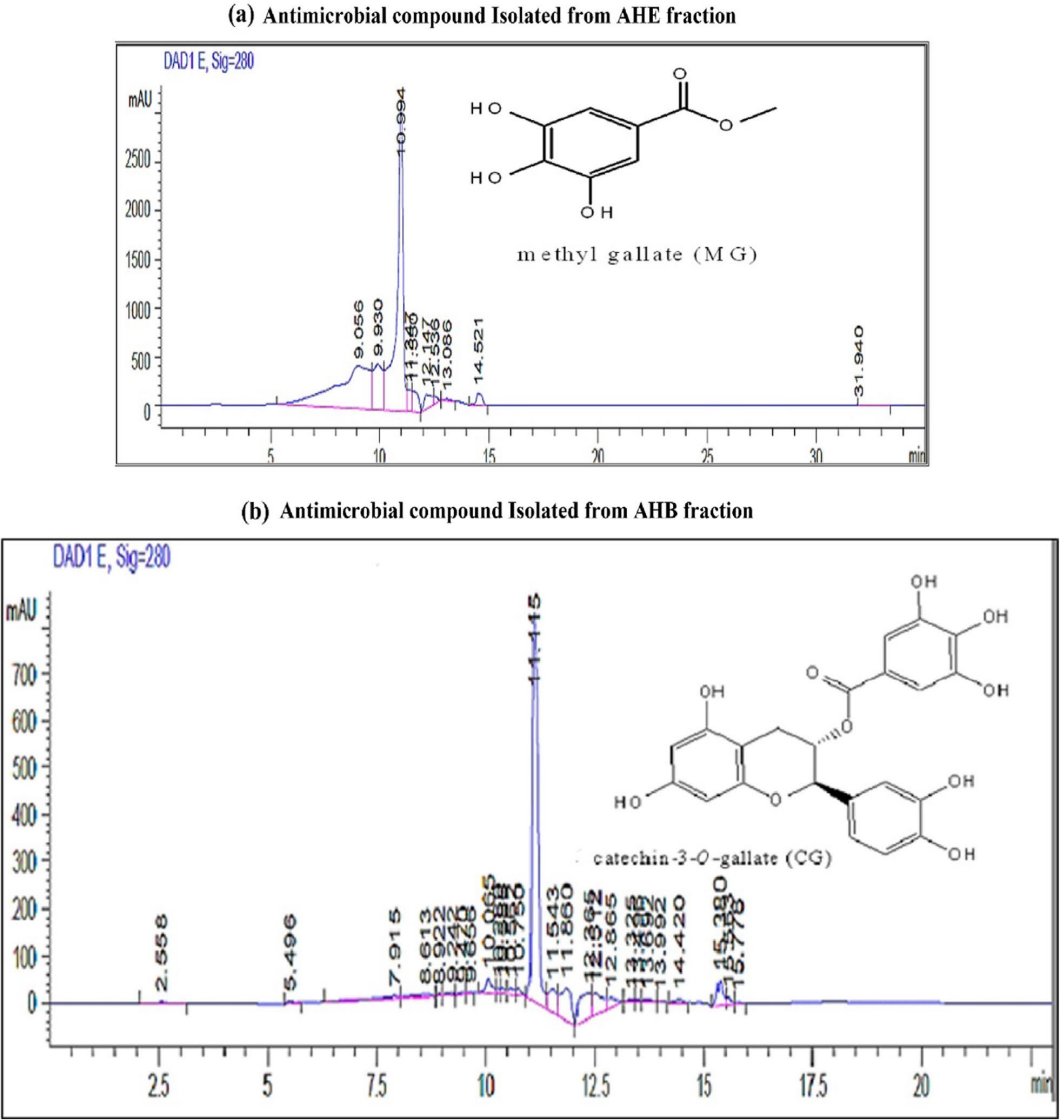
**a** Analytical HPLC chromatogram of methyl gallate showing single peaks at 10.994 min. **b**: Analytical HPLC chromatogram of catechin 3-*O*-gallate (CG) showing single peak at 11.15 min. Chromatographic conditions: Vision Ht C18 column (5 μm; 10 × 250 mm, Agilent USA). Mobile phase A (Millipore H_2_O) and mobile phase B (acetonitrile) in gradients: 0–5 min; 15% B in A (isocratic run), 5–27 min; 15 to 100% B (Gradient mode), 27–32 min; 15% B in A (for column equilibration). Flow rate; 1 ml/min, injection volume 20 µl. Both compounds showed UV maxima at 280 nm (characteristic of polyphenolic compounds)

The purity of active fractions was determined based on the absorption spectrum obtained by analytical RP-HPLC analysis. Absorption spectra of the purified active constituent from AHE and AHB were achieved at 210–600 nm by using a spectrophotometer coupled with a UV-diode array detector (HPLC–DAD). Isolated active components AHE [ISCO-AHE/F3 and AHB [RP-HPLC-AHB/F5] showed single-peak chromatograms. The spectra of the peaks presented an indication of the family of polyphenolic compounds. Figure 1a, b represents the peak chromatograms and chemical structures of the antimicrobial compounds of *A hydaspica* respectively.

### Compound yield

*Acacia hydaspica* ethyl acetate extract (AHE) yields a 37.5 mg/g dry sample of methyl gallate whereas a 29 mg/g dry sample of Catechin-3-*O*-gallate was obtained from AHB.

### Molecular interaction between *S. aureus* cell surface proteins (Atl, ClfA, and FnBP) and Catechin 3-*O*-gallate (CG) and methyl gallate (MG)

**Fig. 2:**
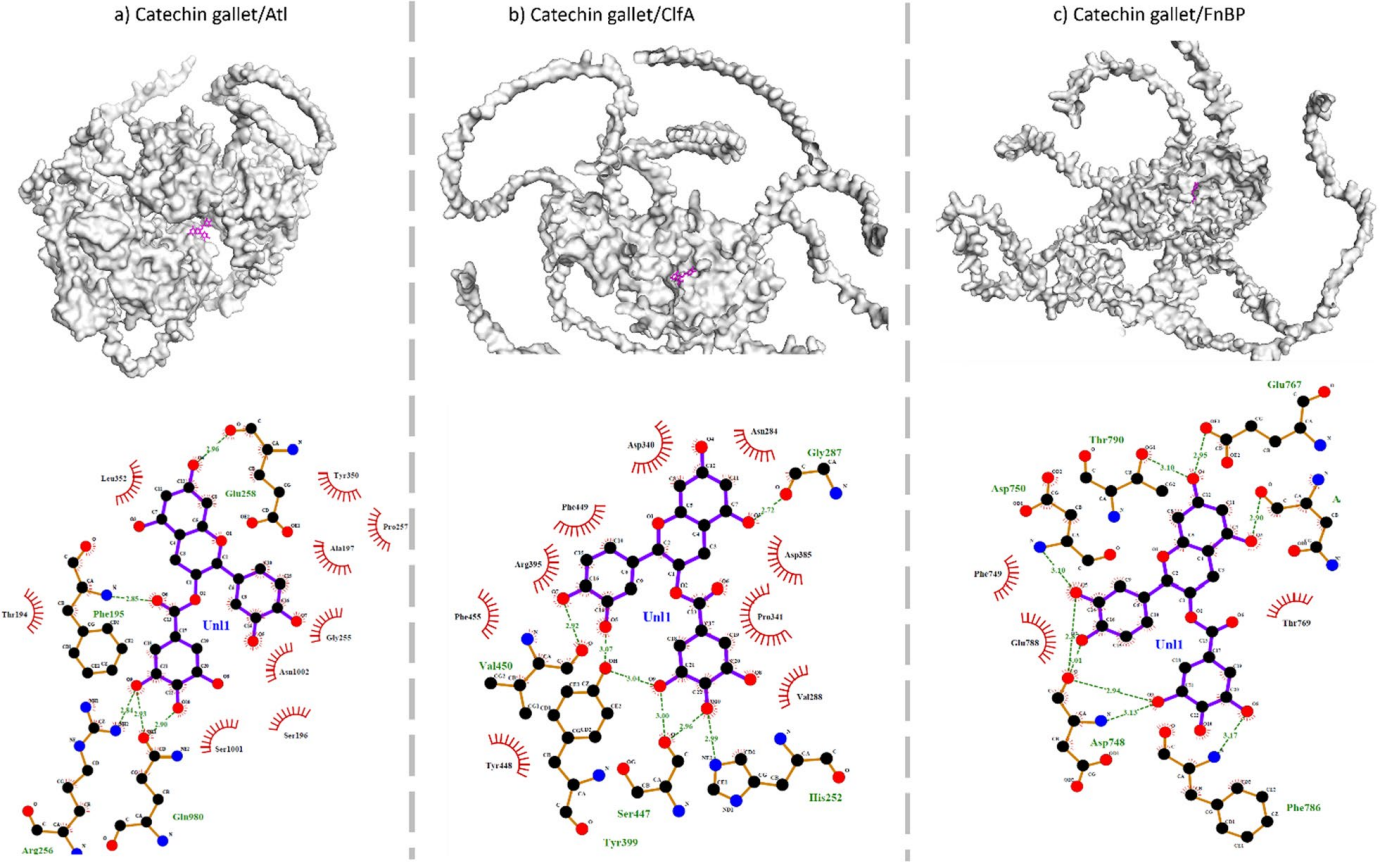
Molecular docked complex of Catechin 3-*O*-gallate with bacterial cell surface proteins. **a** Atl, **b** ClfA, and **c** FnBP. Surface view of protein–ligand complex is shown above where purple colour indicates ligand. Hydrogen bonding is represented with green dotted line. Hydrophobic interactions are shown with red colored spiked semi circles. Purple lined structure indicated ligand

**Fig. 3:**
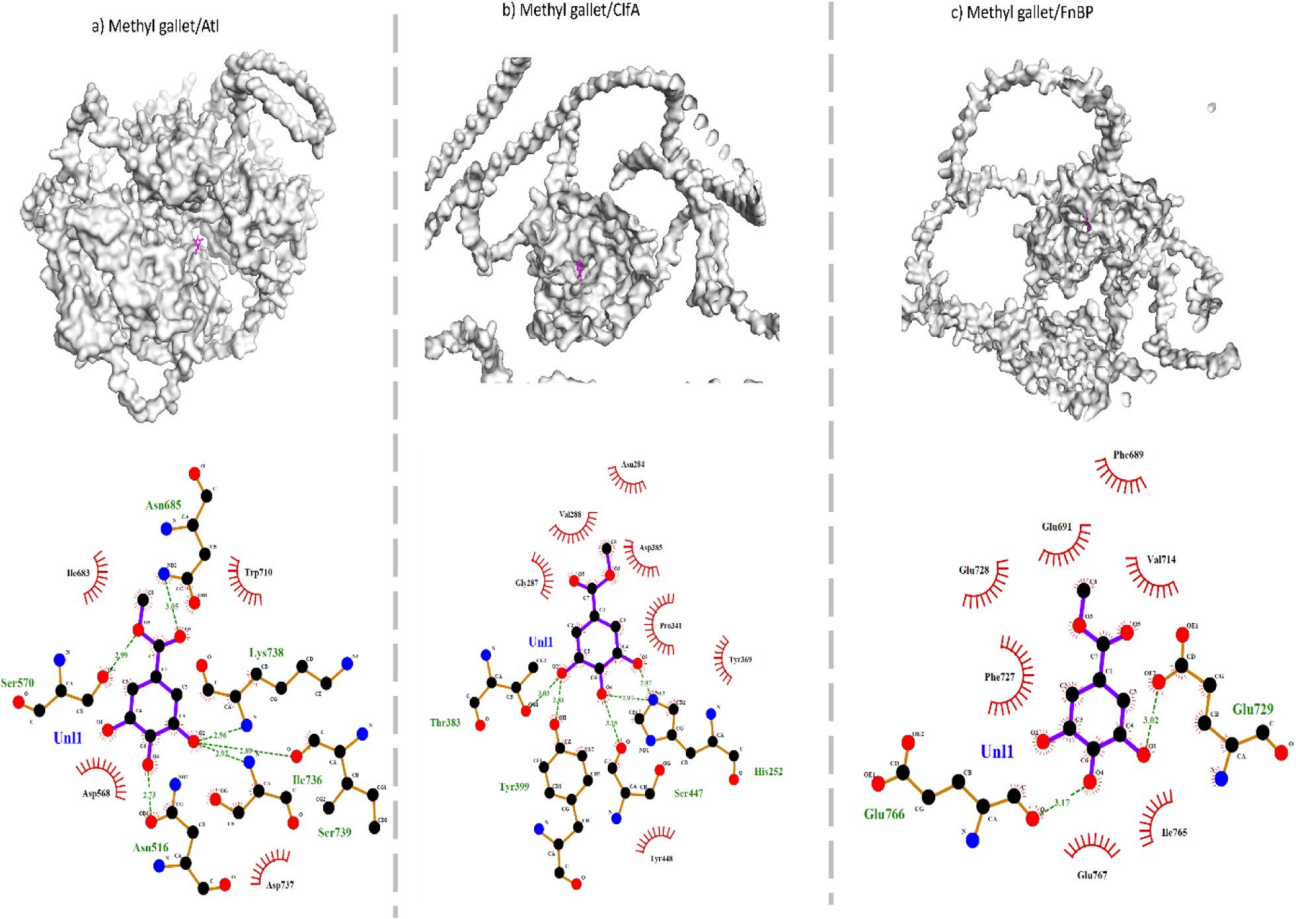
Molecular docked complex of Methyl gallate with bacterial cell surface proteins. **a** Atl, **b** ClfA, and **c** FnBP. Surface view of protein–ligand complex is shown above where purple colour indicates ligand. Hydrogen bonding is represented with green dotted line. Hydrophobic interactions are shown with red colored spiked semi circles. Purple lined structure indicated ligand

Rigid docking analysis revealed that Catechin 3-*O*-gallate made stronger molecular interaction with Atl, ClfA, and FnBP in comparison to methyl gallate (Table 7). CG made a stronger complex with ClfA with a binding energy of − 9.7 relative to Atl (− 8.8) and FnBP (− 8.3). Similarly, MG made a stronger complex with Atl (binding energy − 6.2) relative to FnBP (− 5.6) and ClfA (− 6.1). A molecular interactome study indicated that CG made nine hydrophobic interactions and four hydrogen bonds with Atl (Figs. 2 and 3). Additional file 2: Table S9 showed detailed analysis of docked complexes of bacterial cell surface proteins and methyl gallate and catechin 3-*O*-gallate along with the vina score and cavity size.

**Table 7:** Methyl gallate and catechin 3-*O*-gallate docked complex with bacterial cell surface proteins

Drug	Protein	Vina score
Catechin 3-*O*-gallate	Atl	− 8.8
	ClfA	− 9.7
	FnBP	− 8.3
Methyl gallate	Atl	− 6.2
	ClfA	− 6.1
	FnBP	− 5.6

## Discussion

Currently, antimicrobial resistance is on top among serious health threats due to infections resulting from resistant bacteria. Such types of infections are most prevalent in susceptible patients experiencing cancer chemotherapy, dialysis and surgery. The causes of antibiotic resistance are complex. The development of new and novel antibiotics is the need of the hour. Medicinal plants have been in use since eras for treating different ailments. The universality and usefulness of traditional medicine/medicinal herbs are obvious from their persistent use by a substantial portion of the world’s inhabitants (Cáceres et al. 1995; Gilani et al. 2010; Shinwari and Qaisar 2011). The test organisms used in our investigation were selected based on their association in varied pathologic human infections. For example; *E. coli* causes septicemia and lung infection, gall bladder and skin lesions besides various forms of foodstuff-borne ailments that commonly consequences in diarrhea (Mølbak et al. 2002). *Staphylococcus aureus* infection is a leading reason of skin, bone, joint, soft-tissue, respiratory and endovascular disorders (Lowy 1998). To better assess the efficiency of the natural products, known antibiotics were used for comparison.

The outcomes of the present examination established a general trend of higher antimicrobial activity in ethyl acetate (AHE) and *n-*butanol (AHB) fractions of *A. hydaspica* methanol extract. Hence AHE and AHB fractions showing lower MIC values and significant microbial growth inhibitory potential were selected for bioassay-guided isolation of lead antimicrobial compounds. Extract/fraction showed specificity in activity against different bacterial strains. The ethyl acetate fraction is the most active fraction against both Gram +ive and Gram −ive bacterial strains and showed lower MIC values. Current findings are in line with the outcomes of Guta et al. (2009), illustrating ethyl acetate fraction of *A. nilotica* fruit to possess maximum antibacterial activity. Previous research demonstrated that Gram-negative and Gram-positive bacteria both were found to be very challenging, the non-polar fractions were the only ones to demonstrate potential against both strains of bacteria (Martini and Eloff 1998). On contrary, our results indicate that polar fractions showed higher potential against both Gram-positive and Gram-negative bacteria might be due to the combined influences of various compounds present in each fraction. It may be likely that the polarity of the polar phytochemicals like tannins, flavonoids and alkaloids present in various fractions can act mutually with the conformation of the cell structure of this sort of bacteria. Related antimicrobial findings of methanol extract/fractions accredited antimicrobial potential to bioactive phytochemicals were reported in earlier studies (Negi and Dave 2010). Analogous results were obvious from the study of Mbolekwa et al. (2014), demonstrating the antibacterial activity of various fractions of stem bark extracts of *A. mearnsii* De Wild, and results of their study reveal that ethyl acetate fraction was the maximum active fraction against all tested bacterial strains. Mattana et al. (2010) study indicated that the antimicrobial activity of *A. aroma* against methicillin-resistant and methicillin-sensitive *Staphylococcus* is due to the presence of flavonoids. Flavonoids are potential antimicrobial substances possibly due to their ability to interact with soluble and extracellular proteins and to formulate bonding with the cell wall of microbes and thus stimulating bacterial growth inhibition (Cowan 1999).

Antifungal activity testing indicated that *A. hydaspica* has potent activity against *A. fumigatus* strain and *A. solani* while moderate activity against *A. niger* and *A. flavus*. Among the tested fractions, AHE seems to be best potent fraction against all tested strains, inhibit significantly the growth of *F. solani* and *A. niger*; whereas moderately inhibits the growth of *A. flavus* and *A. fumigatus.* AHB showed inhibitory potential only against *F. solani* and moderately inhibit its growth while non-significant inhibition was observed against other strains. The varied potency of various fractions of *A. hydaspica* against tested fungal strains confers a diversity of phytochemicals in different fractions that results in variable/specific potency of activity. Among all tested samples from *A. hydaspica* ethyl acetate fraction presented the uppermost antifungal activity against all tested strains. Physiologically active flavonoid constituents in AHE and AHM might be responsible for the antifungal activity. Studies point out that flavonoids signify novel leads, and forthcoming research may permit the advancement of pharmacologically suitable antimicrobial mediators or classes of agents (Cushnie and Lamb 2005). The antifungal activity of flavonoids is due to their ability to make bonding with extracellular and soluble proteins as well as with fungal cell walls. The highly lipophilic nature of flavonoids helps in disrupting the fungal membrane (Arif et al. 2009). Due to the pervasive attitude of flavonoids to impede spore germination of plant pathogens, they have been anticipated to be operative against human fungal pathogens (Harborne and Williams 2000).

To isolate the active metabolites with antimicrobial activity from AHE and AHB. Both fractions were subjected to bioassay-guided isolation of lead compounds responsible for the observed antimicrobial activity. The AHE fraction showed significant potential against specific bacterial (gram-positive and gram-negative) as well as fungal strains. Both AHE and AHB fractions possess modest antibacterial activity against various bacterial and fungal strains, the lowest activity was recorded in AHB fractions. Bioassay-guided isolation leads to the isolation of lead antimicrobial compounds from AHE and AHB. Structures of purified compounds were elucidated by 1D and 2D NMR and mass spectrometric analysis (ESI and APCI). Details of NMR data have been published in our previous investigations of the antioxidant activity of compounds (Afsar et al. 2018). The compounds isolated from *A. hydaspica* were identified by comparison of the physical data with those reported previously (Nonaka et al. 1983; Razak et al. 2019). Data indicated that ISCO-AHE/F3 was a simple polyphenol methyl-gallate (MG) (Nishioka et al. 1998), whereas the active component from AHB (RP-HPLC-AHB/F5) was identified as Catechin-3-*O*-gallate (CG) (Min-Won et al. 1992). MG showed significant antibacterial activity against *E. coli* (MIC_50_ = 21.5 µg/ml), *B. subtilus* (MIC_50_ = 23 µg/ml) and *S. aureus* (MIC_50_ = 39.1 µg/ml) while moderate to low activity was noticed against other tested bacterial strains. A study by Mashram, Mahesh and Satish reported the anti-microbial activity of *A. nilotica* against *S. aureus, B. subtilis* and *E. coli.* The leaf and bark extracts showed zone of inhibition ranging from 7.5–16 and 8–15.5 mm respectively and the *E. coli* strain seems to be more susceptible (Mahesh and Satish 2008; Mashram 2009). Similarly, in the current investigation, we observed that the *E. coli* strain was more susceptible to AHE and MG isolated from AHE. Furthermore, antifungal testing reveals that MG showed potent antifungal activity against *F. solani* (MIC_50_ = 33.9 µg/ml) and *A. niger* (MIC_50_ = 41.5 µg/ml) while lower antifungal activity was seen in other tested strains. AHB fractions and pure compound (CG) showed specific antibacterial activity against *S. aureus* only while compound and enriched fractions showed moderate to no activity against other bacterial and fungal strains respectively. Catechin 3-*O*-gallate (CG) showed higher inhibitory potential against S. aureus with MIC_50_ 10.1 µg/ml in comparison to Methyl gallate (MG). However overall MG is a more effective antimicrobial compound isolated from *A. hydaspica* as it showed inhibitory potential against various strains. A study by Ahmed and coworkers (2017) indicated the potential inhibitory efficacy of MG against MRSA-resistant *S. aureus* (MIC of 50 µg/ml). It is well acquainted that green tea is a major source of catechins and investigations provide clear proof that the catechin compounds (EGC, EGCg and ECg) of green tea constitute its antibacterial activity (Bidlack 2001; Taylor et al. 2005). However, the effectivity of the individual components from green tea was rather moderate. Stapleton and coworkers tested a range of natural and synthetic catechins and catechin gallates against MRSA and observed that they possessed either inadequate effectiveness or no noticeable activity; MICs of compounds were extended from 64 to > 256 mg/l (Stapleton et al. 2004). Contrary to these findings we observe that catechin compound (CG) isolated from AHB showed significant activity (10.1 µg/ml) against *S. aureus* however the potency of CG and other enriched fractions was lowest to no obvious action against other strains. MG isolated from AHE showed significant potential against *S. aureus* as well (39.1 µg/ml). These outcomes are noteworthy as *S. aureus* is among the major sources of both nosocomial and communal-acquired infections globally (Taylor et al. 2005). Whilst these were not efficient agents for systemic use as established antibacterial agents, current analysis reveals that they may be applied as topical agents for the cure of external microbial infections. The improved activity of CG isolated from *A. hydaspica* compared to green tea catechins might be due to varied substitution of the gallate moiety of catechin (Stapleton et al. 2004). The antimicrobial action of methyl gallate (MG) compound isolated from *A. hydaspica* against various bacterial and fungal pathogens involved in various diseases specified the pharmacological importance of the plant. Sanchez and colleagues (2013) identified Methyl gallate as the major antimicrobial agent from *A. farnesiana*, similarly, we identified methyl gallate as the major antimicrobial compound from *A. hydaspica as its e*ffectiveness against various bacterial and fungal strains. The antimicrobial activity of methyl gallate validates the antimicrobial activity of *A. hydaspica* extracts and partially supports its various traditional medicinal purposes. Present research offers the basis for the use of *A. hydaspica* methanol extract and its different fractions for the cure of various infections related to the microbes under research. Furthermore, it’s interesting to find out that besides green tea *A. hydaspica* appears to be a subtle source of catechins and its gallate moieties are the source to be additionally responsible for observed antibacterial activity. Predominant in vitro data approved the significance of catechins in disease management in humans. Catechin-facilitated photo-protection of human skin to counter bacterial infections (Hsu 2005). The observed antimicrobial effect of AHE and AHB fractions against various strains and specific action of isolated compounds indicated that the observed effect might be due to the synergistic effect of various components in *A. hyaspica*. However, targeting specific microbial agent-isolated compounds would be more efficient. A lot of investigations have been done to elucidate the mechanisms behind the antimicrobial action of plant phytoconstituents but still, it is less understood. Flavonoids may inhibit cytoplasmic membrane function, DNA gyrase, and β-hydroxy-acyl carrier protein dehydratase functions (Cushnie and Lamb 2005; Zhang et al. 2008). Despite this plethora of observations on the multifaceted nature of the antibacterial effects of catechin and MG, there is little published data on the mechanisms of action of these agents. Studies on the mechanism of antibacterial action of catechins revealed that catechins do not gain entry into cells but exert their effects from the cell membrane. These observations suggest that membrane interactions of catechin are governed by the degree of hydroxylation of the B-ring, the presence of a gallate moiety and the stereochemistry of the C-ring. Whereas investigations on MG concluded that it exhibits antimicrobial activity by damaging the membrane integrity, causing physiological changes leading to a significant reduction in the pH, affecting the membrane potential, and decreasing cellular ATP levels.

Bioinformatics approaches have revolutionized the process of drug design. Molecular docking approaches have further facilitated the delineation of the appropriate molecular compound for targeting the molecular player. In the current study, the efficacy of Catechin 3-*O*-gallate (CG) and Methyl gallate (MG) was accessed to target *Staphylococcus aureus* cell surface proteins, Autolysins (Atl), Clumping factor A (ClfA), and fibronectin-binding protein (FnBP). These proteins promote the adhesion and proliferation of *S. aureus* and induce its pathogenicity (Foster 2019). Previously, the activity of epicatechin-3-*O*-gallate-(4β,8)-epicatechin-3′-*O*-gallate against the adhesion capacity of Porphyromonas gingivalis was evaluated through molecular docking and in vitro testing. Molecular docking analysis revealed the anti-adhesive activity of epicatechin-3-*O*-gallate-(4β,8)-epicatechin-3′-*O*-gallate (Schmuch et al. 2015). Based on high binding affinity, larger cavity size, and molecular interactome analysis, it was found in the present study that CG made a stronger complex with cell surface proteins than MG. Furthermore, CG interacted more strongly with ClfA in comparison to Atl and FnBP. Studies indicated that ClfA is a virulence factor that allows *S. aureus* to bind with plasma protein fibrinogen, furthermore, ClfA promotes the colonization of *S. aureus*. Molecular docking investigation indicated that inhibition of ClfA through CG might be a good therapeutic strategy to target *S. aureus*. Additional experiments are underway to provide information regarding other physiological sites of relevance that may be affected in this micro-organism, especially concerning the search for inhibitors of different virulence factors.

Future investigations should focus on the use of methyl gallate and catechin 3-*O*-gallate in combination with standard antibiotics to get insights into the synergistic value of these compounds. Both compounds showed efficient inhibitory potential against specific microbial strains comparable to standard antibiotics. They could be an alternative treatment option to antibiotics or a supplement to antibiotics, perhaps reducing the risk of antibiotic resistance. More studies are necessary to demonstrate the efficacy of these medical treatments and the safety of their use.

## Supplementary Information

**Additional file 1: Figure S1.** Flowchart summarization of the bioassay guided fractionation and compound isolation. **Figure S2.** ISCO chromatogram showing the step gradient run and pattern of spectral peaks, yellow lines indicates the pooling arrangement of fractions. **Figure S3.** Analytical HPLC Chromatogram of Sp-AHB/F4 at 280 nm. Method: 0 min–5 min; 15% B in 85% A (isocratic run), 5–25 min; up to 50% B in 50% A, 25–30 min; upto 100% B, 30.1–35 min; 15% B in 85% A (isocratic run). **Figure S4.** Analytical HPLC Chromatogram of Sp-AHB/F4 at various wavelengths. Method: 0 min–5 min; 15% B in 85% A (isocratic run), 5–25 min; up to 50% B in 50% A, 25–30 min; upto 100% B, 30.1–35 min; 15% B in 85% A (isocratic run). **Figure S5.** Semi-prep RP-HPLC chromatograph of Sp-AHB/F4 fraction indicating the partitioning of fractions according to the spectral peaks. Method: 0 min–5 min; 15% B in 85% A (isocratic run), 5–25 min; up to 70% B in 30% A, 25–27 min; up to 100% B, 27.1–32 min; 15% B in 85% A (isocratic run). **Figure S6.** 1H NMR spectrum of Methyl gallate (MG). Solvent: acetone-d6, Frequency (MHz): 599.67, Nucleus: H, Temperature: 25 °C, Pulse sequence: s2pul, Acquisition time (sec): 1.704, Number of transits: 16, Original point count: 16,384, Spectrum offsets (Hz): 3598.0154, Spectrum type: Standard, sweep width (Hz):9615.4. **Figure S7.** 13C NMR spectrum of Methyl gallate (MG). **Figure S8.** 1H NMR spectrum of Catechin 3-*O*-gallate (CG). Solvent: methanol-d4, Frequency (MHz): 599.67, Nucleus: H, Temperature: 25 °C, Pulse sequence: s2pul, Acquisition time (sec): 1.7039, Number of transits: 32, Original point count: 16,384, Spectrum offsets (Hz): 3598.0154, Spectrum type: Standard, sweep width (Hz): 9615.38. **Figure S9.** 13C NMR spectrum of Catechin 3-*O*-gallate (CG).

**Additional file 2: Table S1.** Antibacterial activity of VLC-AHE fractions (Percent growth inhibition). **Table S2.** Antifungal activity of VLC-AHE fractions (Percent Growth Inhibition). **Table S3.** Antibacterial activity of *A. hydaspica*: Isolated fractions/compounds from AHE by flash chromatography (ISCO). **Table S4.** Antifungal activity of *A. hydaspica*: Isolated fractions/compounds from AHE by flash chromatography (ISCO). **Table S5.** Antibacterial activity of *A. hydaspica* isolated fractions from AHB by Sephadex LH20 chromatography against *S. aureus*. **Table S6.** Antibacterial activity of *A. hydaspic*a isolated fractions from AHB by Semi-prep RP-HPLC against *S. aureus*. **Table S7.** ^1^H NMR data of antimicrobial compounds isolated from *A. hydaspica* (Coupling constant J in Hertz). **Table S8.** ^13^C NMR data of antimicrobial compounds isolated from *A. hydaspica*. **Table S9.** Docked complexes of bacterial cell surface proteins and methyl gallate and catechin 3-*O*-gallate along with the vina score and cavity size.

## Data Availability

The datasets used and/or analyzed during the current study are available from the corresponding author on reasonable request.

## Abbreviations

NMR: Nuclear magnetic resonance
RP-HPLC: Reverse phase high-pressure liquid chromatography
APCI: Atmospheric pressure chemical ionization
ESI–MS: Electrospray ionization mass spectrometry
HMBC: Heteronuclear multiple-bond coherence
1D and 2D: 1 Dimensional and 2 dimensional
MeOH: Methanol
DMSO: Dimethyl sulfoxide
Atl: Autolysins
ClfA: Clumping factor A
FnBP: Fibronectin-binding protein
AHE: Acacia hydaspica ethyl acetate fraction
AHB: Acacia hydaspica butanol fraction
EGC: Epigallocatechin
