# Supplementary material for: Bioassay-guided isolation and characterization of lead antimicrobial compounds from Acacia hydaspica plant extract
Source: AMB Express. 2022 Dec 15;12:156. doi: 10.1186/s13568-022-01501-y (PMC9755427; doi:10.1186/s13568-022-01501-y)
Supplement: Supplementary file 1 — Additional file 1: Figure S1. Flowchart summarization of the bioassay guided fractionation and compound isolation. Figure S2. ISCO chromatogram showing the step gradient run and pattern of spectral peaks, yellow lines indicates the pooling arrangement of fractions. Figure S3. Analytical HPLC Chromatogram of Sp-AHB/F4 at 280 nm. Method: 0 min–5 min; 15% B in 85% A (isocratic run), 5–25 min; up to 50% B in 50% A, 25–30 min; upto 100% B, 30.1–35 min; 15% B in 85% A (isocratic run). Figure S4. Analytical HPLC Chromatogram of Sp-AHB/F4 at various wavelengths. Method: 0 min–5 min; 15% B in 85% A (isocratic run), 5–25 min; up to 50% B in 50% A, 25–30 min; upto 100% B, 30.1–35 min; 15% B in 85% A (isocratic run). Figure S5. Semi-prep RP-HPLC chromatograph of Sp-AHB/F4 fraction indicating the partitioning of fractions according to the spectral peaks. Method: 0 min–5 min; 15% B in 85% A (isocratic run), 5–25 min; up to 70% B in 30% A, 25–27 min; up to 100% B, 27.1–32 min; 15% B in 85% A (isocratic run). Figure S6. 1H NMR spectrum of Methyl gallate (MG). Solvent: acetone-d6, Frequency (MHz): 599.67, Nucleus: H, Temperature: 25 °C, Pulse sequence: s2pul, Acquisition time (sec): 1.704, Number of transits: 16, Original point count: 16,384, Spectrum offsets (Hz): 3598.0154, Spectrum type: Standard, sweep width (Hz):9615.4. Figure S7. 13C NMR spectrum of Methyl gallate (MG). Figure S8. 1H NMR spectrum of Catechin 3-O-gallate (CG). Solvent: methanol-d4, Frequency (MHz): 599.67, Nucleus: H, Temperature: 25 °C, Pulse sequence: s2pul, Acquisition time (sec): 1.7039, Number of transits: 32, Original point count: 16,384, Spectrum offsets (Hz): 3598.0154, Spectrum type: Standard, sweep width (Hz): 9615.38. Figure S9. 13C NMR spectrum of Catechin 3-O-gallate (CG). [file 13568_2022_1501_MOESM1_ESM.docx]

**Additional file S1**

**Title: Bioassay guided isolation and characterization of lead antimicrobial compounds from *Acacia hydaspica* plant extract.**

Tayyaba afsar^1^, Suhail Razak^1*^, Ali Almajwal^1^, Maria Shabbir^2,^ Khushbukhat Khan^2,^ Janeen Trembly^3,4,5^, Nawaf W. Alruwaili^1^.


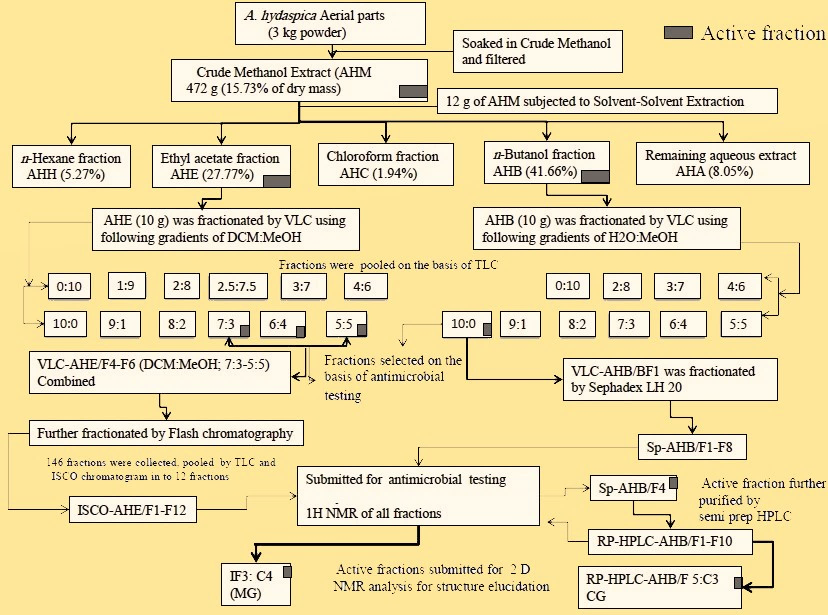


Figure S1: Flowchart summarization of the bioassay guided fractionation and compound isolation.


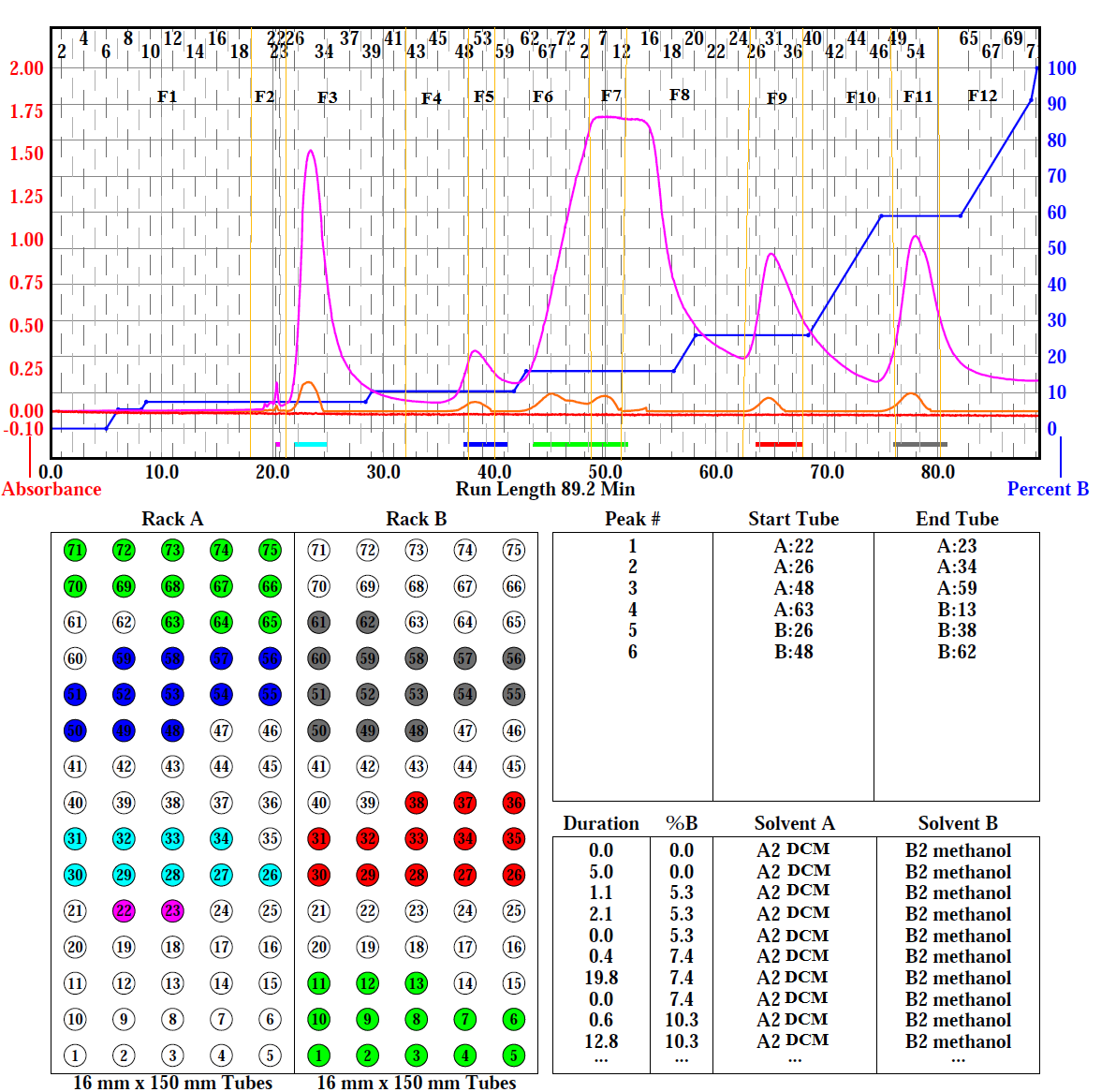


Figure 2: ISCO chromatogram showing the step gradient run and pattern of spectral peaks, yellow lines indicates the pooling arrangement of fractions.


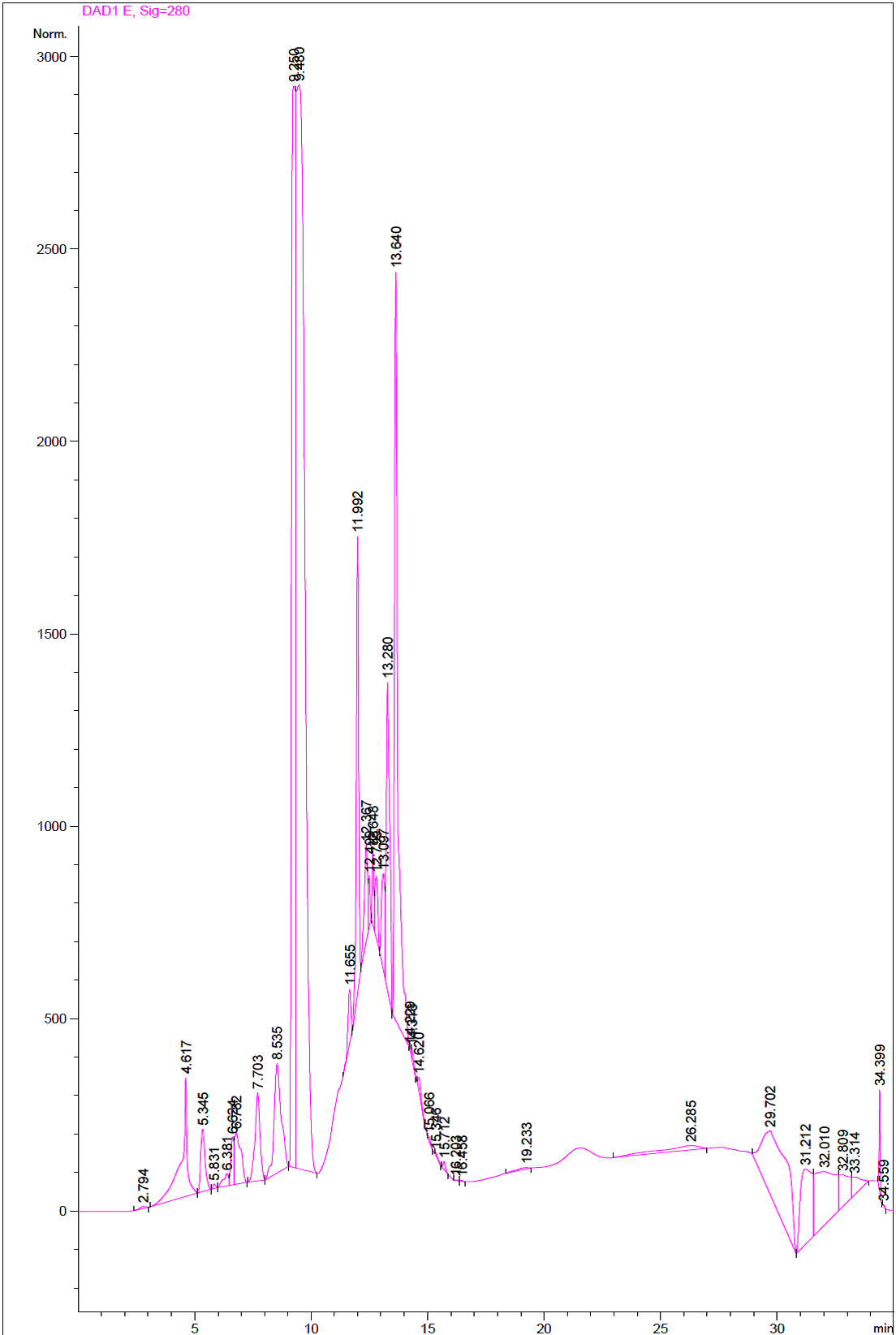


Figure S3: Analytical HPLC Chromatogram of Sp-AHB/F4 at 280nm. Method: 0 min-5min; 15% B in 85% A (isocratic run), 5–25min; up to 50% B in 50% A, 25-30min; upto 100% B, 30.1-35min; 15% B in 85% A (isocratic run).


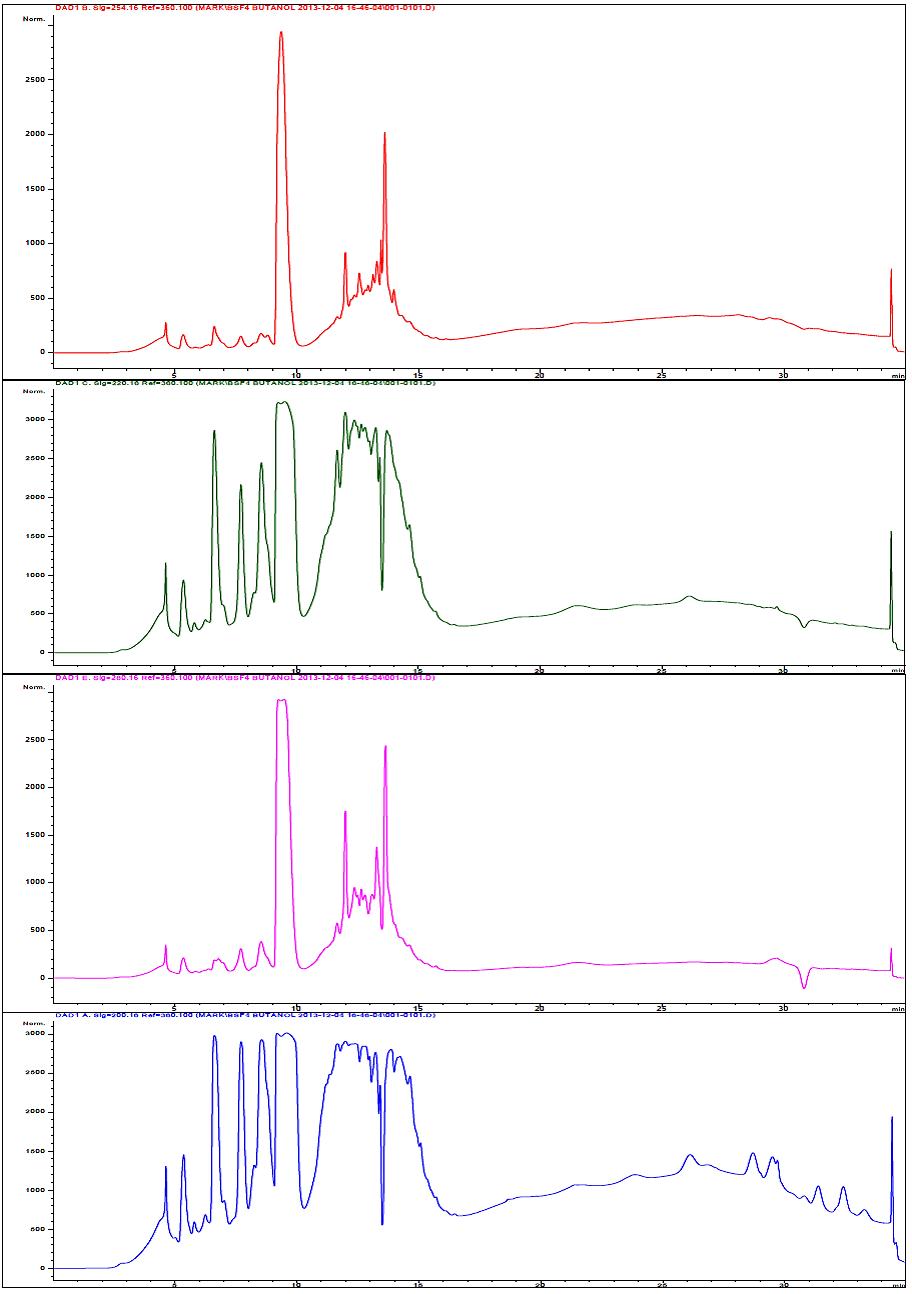


Figure S4: Analytical HPLC Chromatogram of Sp-AHB/F4 at various wavelengths. Method: 0 min-5min; 15% B in 85% A (isocratic run), 5–25min; up to 50% B in 50% A, 25-30min; upto 100% B, 30.1-35min; 15% B in 85% A (isocratic run).


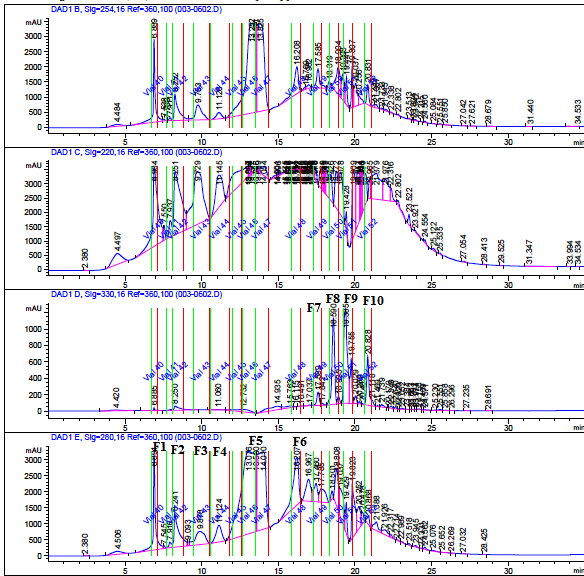


Figure S5: Semi-prep RP-HPLC chromatograph of Sp-AHB/F4 fraction indicating the partitioning of fractions according to the spectral peaks. Method: 0 min - 5min; 15% B in 85% A (isocratic run), 5 - 25 min; up to 70% B in 30% A, 25 - 27min; up to 100% B, 27.1 - 32 min; 15% B in 85% A (isocratic run).


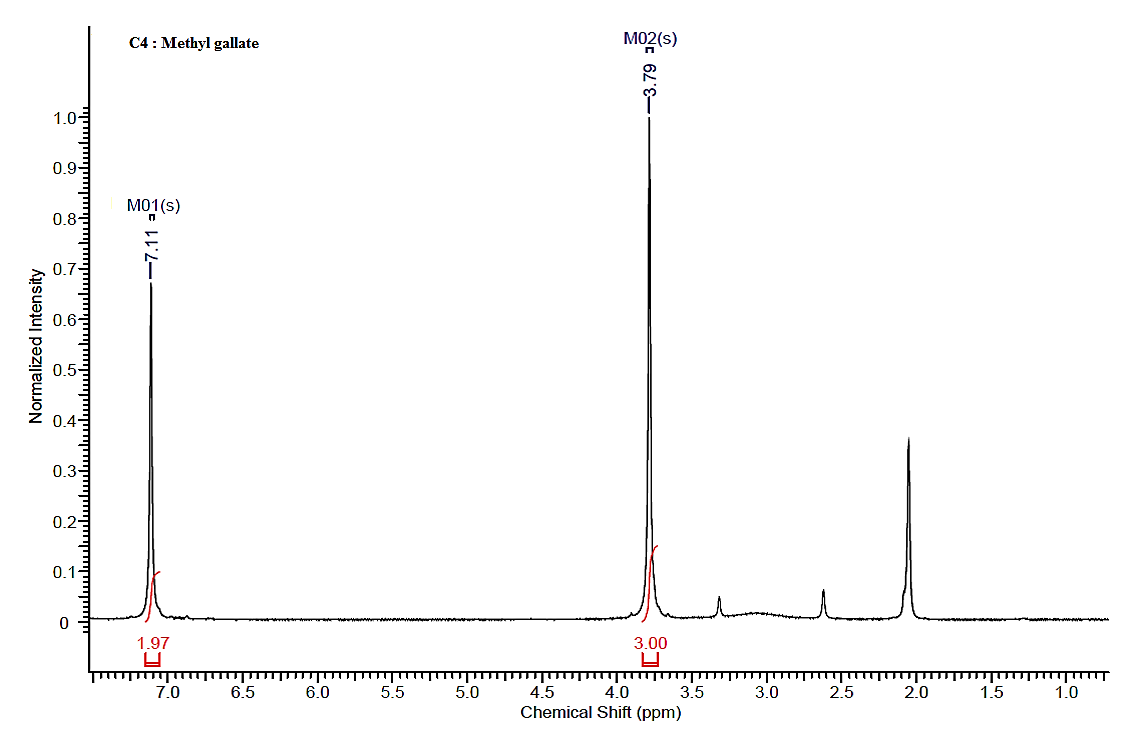
**Figure S6:** 1H NMR spectrum of Methyl gallate (MG). Solvent: acetone-d6, Frequency (MHz):599.67, Nucleus: H, Temperature: 25^o^C, Pulse sequence: s2pul, Acquisition time (sec):1.704, Number of transits: 16, Original point count: 16384, Spectrum offsets (Hz): 3598.0154, Spectrum type: Standard, sweep width (Hz):9615.4.

**
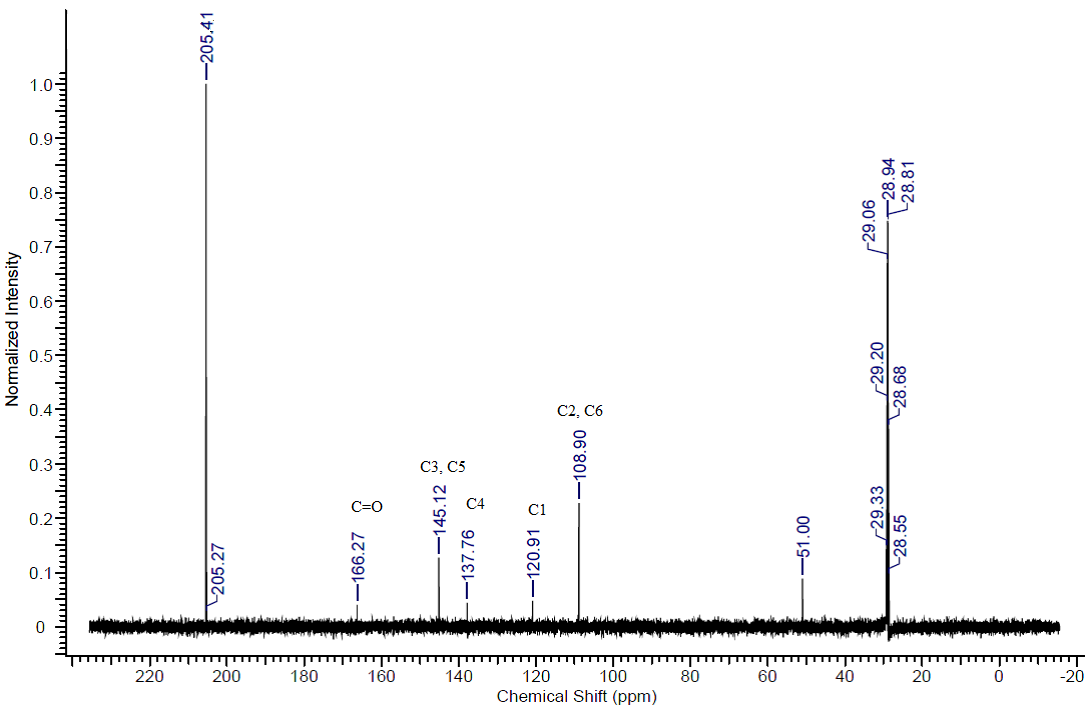
**

**Figure S7**: 13C NMR spectrum of Methyl gallate (MG).


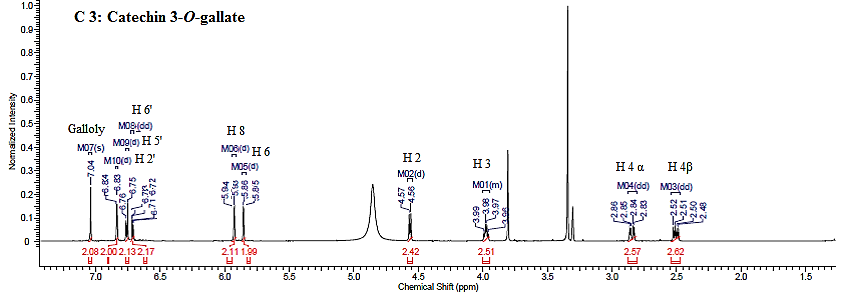


Figure S8: 1H NMR spectrum of Catechin 3-*O*-gallate (CG). Solvent: methanol-d4, Frequency (MHz):599.67, Nucleus: H, Temperature: 25^o^C, Pulse sequence: s2pul, Acquisition time (sec):1.7039, Number of transits: 32, Original point count: 16384, Spectrum offsets (Hz): 3598.0154, Spectrum type: Standard, sweep width (Hz):9615.38.

**
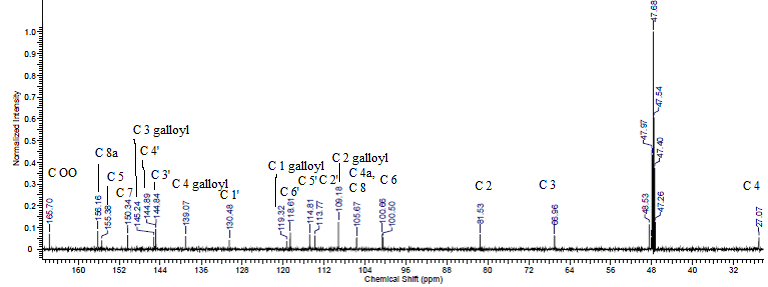
**

**Figure S9**: 13C NMR spectrum of Catechin 3-*O*-gallate (CG).
