# Supplementary material for: Bioassay-guided isolation and characterization of lead antimicrobial compounds from Acacia hydaspica plant extract
Source: AMB Express. 2022 Dec 15;12:156. doi: 10.1186/s13568-022-01501-y (PMC9755427; doi:10.1186/s13568-022-01501-y)
Supplement: Supplementary file 2 — Additional file 2: Table S1. Antibacterial activity of VLC-AHE fractions (Percent growth inhibition). Table S2. Antifungal activity of VLC-AHE fractions (Percent Growth Inhibition). Table S3. Antibacterial activity of A. hydaspica: Isolated fractions/compounds from AHE by flash chromatography (ISCO). Table S4. Antifungal activity of A. hydaspica: Isolated fractions/compounds from AHE by flash chromatography (ISCO). Table S5. Antibacterial activity of A. hydaspica isolated fractions from AHB by Sephadex LH20 chromatography against S. aureus. Table S6. Antibacterial activity of A. hydaspica isolated fractions from AHB by Semi-prep RP-HPLC against S. aureus. Table S7. 1H NMR data of antimicrobial compounds isolated from A. hydaspica (Coupling constant J in Hertz). Table S8. 13C NMR data of antimicrobial compounds isolated from A. hydaspica. Table S9. Docked complexes of bacterial cell surface proteins and methyl gallate and catechin 3-O-gallate along with the vina score and cavity size. [file 13568_2022_1501_MOESM2_ESM.docx]

**Additional file S2**

**Title: Bioassay guided isolation and characterization of lead antimicrobial compounds from *Acacia hydaspica* plant extract**

Tayyaba afsar^1^, Suhail Razak^1*^, Ali Almajwal^1^, Maria Shabbir^2,^ Khushbukhat Khan^2,^ Janeen Trembly^3,4,5^, Nawaf W. Alruwaili^1^.

Table S1: Antibacterial activity of VLC-AHE fractions (Percent growth inhibition).

| Samples | *S.*  *aureus* | *E. coli* | *P.*  *aeruginosa* | *E.*  *faecalis* | *K.*  *pneumoniae* | *A.*  *baumannii* | *B.*  *subtilis* |
| --- | --- | --- | --- | --- | --- | --- | --- |
| AHE/F1 | -12 | -4 | -7 | -9.6 | -22 | -3 | 26 |
| AHE/F2 | 7 | 4 | 3 | -5.7 | -11 | -12 | 18 |
| AHE/F3 | 9 | 19 | 9 | 2.6 | -3 | -28 | 16 |
| AHE/F4 | 46 | 54 | 22 | 13.2 | 10 | 7 | 70 |
| AHE/F5 | 43 | 58 | 27.1 | 11.7 | 8 | 9 | 65 |
| AHE/F6 | 40 | 55 | 25.5 | 15.2 | 9.5 | 8 | 69 |
| AHE/F7 | 3 | 10 | 10 | 7.1 | -2 | -2 | 38 |
| AHE/F8 | -36 | 16 | 11 | -4 | -2 | -5 | 22.1 |
| AHE/F9 | 10 | 12 | 12 | 12 | -10 | 1 | 25.6 |
| AHE/F10 | -4 | 10 | -16 | 11 | -24 | 4 | 34 |
| AHE/F11 | 7 | 10 | 5 | 3 | -14 | -3 | 30.1 |
| AHE/F12 | 9 | 15 | 3 | 7 | -10 | -12 | 33.6 |
| Tetracyclin | 94 | 95 | 95 | - | 93 | 95 | 96 |
| Penicillin G | - | - | - | 94 | - | - | - |

Table S2: Antifungal activity of VLC-AHE fractions (Percent Growth Inhibition)

| VLC fractions/antibiotic | *C.*  *neoformans* | *C.*  *albicans* |  | *F.*  *Solani* | *A.*  *niger* |
| --- | --- | --- | --- | --- | --- |
| AHE/F1 | -4.52 | 4.1 |  | 12.1 | -10.6 |
| AHE/F2 | -5.51 | 5.13 |  | 13 | -7.7 |
| AHE/F3 | -3.62 | 11.3 |  | 9.6 | 8.6 |
| AHE/F4 | 11.29 | 34.7 |  | 75.6 | 63.2 |
| AHE/F5 | 10.92 | 32.7 |  | 73.6 | 71.7 |
| AHE/F6 | 9.35 | 31.6 |  | 75.5 | 65.2 |
| AHE/F7 | -2.89 | 10.1 |  | 10.5 | 9.1 |
| AHE/F8 | 4.88 | 12.8 |  | 14.5 | 14 |
| AHE/F9 | 2.31 | 12.1 |  | 16.9 | 12.9 |
| AHE/F10 | 4.41 | 1.2 |  | -16 | 16.2 |
| AHE/F11 | 2.36 | 5.3 |  | 5.6 | 3.9 |
| AHE/F12 | 1.66 | -20.9 |  | 3.6 | 12.1 |
| Amphotericin B | 94.9 | 82.70 |  | 90.1 | 92.4 |

Table S3: Antibacterial activity of *A. hydaspica*: Isolated fractions/compounds from AHE by flash chromatography (ISCO)

| *Microorganism* | | | | | | | | | | | | | | | |  |
| --- | --- | --- | --- | --- | --- | --- | --- | --- | --- | --- | --- | --- | --- | --- | --- | --- |
|  | ***Gram + ive*** | | | | | |  | ***Gram – ive*** | | | | | | | | |
| Samples | ***S.***  ***aureus*** | | ***E.***  ***faecalis*** | | ***B.***  ***Subtilis*** | |  | ***E.***  ***Coli*** | | ***P.***  ***aeruginosa*** | | ***K.***  ***pneumoniae*** | | ***A.***  ***baumannii*** | | |
|  | **MIC_50_** | **%**  **GI** | **MIC_50_** | **%**  **GI** | **MIC_50_** | **%**  **GI** |  | **MIC_50_** | **%**  **GI** | **MIC_50_** | **%**  **GI** | **MIC_50_** | **%**  **GI** | **MIC_50_** | **%**  **GI** | |
| AHE/F1 | _ | -14 | _ | -2 | _ | 2 |  | _ | -1 | _ | 6 | _ | -11 | _ | -11 | |
| AHE/F2 | _ | -10 | _ | 5 | _ | 22 |  | _ | 21 | _ | 9 | _ | -3 | _ | -4 | |
| AHE/F3 | 39.1 | 91 | _ | 44 | 23 | 90 |  | 21.5 | 99 | _ | 46 | _ | 31 | _ | 25 | |
| AHE/F4 | _ | -17 | _ | 11 | _ | 41 |  | _ | 36 | _ | 13 | _ | 10 | _ | 5 | |
| AHE/F5 | _ | 21 | _ | 18 | _ | 46 |  | 151.2 | 62 | _ | 15 | _ | 8 | _ | 11 | |
| AHE/F6 | _ | -10 | _ | -3 | _ | 31 |  | _ | 5 | _ | 4 | _ | 3.5 | _ | -1 | |
| AHE/F7 | _ | 24 | _ | 16 | 59.3 | 71 |  | _ | 24 | _ | 1 | _ | 6.6 | _ | -3 | |
| AHE/F8 | _ | 3 | _ | 5 | _ | 18 |  | _ | 3 | _ | -3 | _ | -4 | _ | -6 | |
| AHE/F9 | _ | 2 | _ | 7 | _ | 43 |  | _ | 48 | _ | 11 | _ | -3 | _ | -12 | |
| AHE/F10 | _ | -10 | _ | -2 | _ | 8 |  | _ | 16 | _ | 9 | _ | -14 | _ | 6 | |
| AHE/F11 | _ | 25 | _ | 26 | _ | 13 |  | _ | 36 | _ | -6 | _ | -6 | _ | -14 | |
| AHE/F12 | _ | -11 | _ | -7 | _ | -16 |  | _ | -3 | _ | -5 | _ | -11 | _ | -5 | |
| Tetracycline | _ | 95 | _ | _ | _ | 99 |  | _ | 96 | _ | 94 | _ | 95 | _ | 96 | |
| Penicillin G | _ | _ | _ | 94 | _ | _ |  | _ | _ | _ | _ | _ | _ | _ | _ | |

-; not done, MIC_50_ (Minimum inhibitory concentration resulting in 50% growth inhibition in µg/ml) and GI (growth inhibition at 200 µg/ml)

Table S4: Antifungal activity of *A. hydaspica*: Isolated fractions/compounds from AHE by flash chromatography (ISCO)

|  | *Microorganisms* | | | | | | | |
| --- | --- | --- | --- | --- | --- | --- | --- | --- |
|  | ***Yeast*** | |  | | ***Fungi*** | |  | |
| Samples | ***C. neoformans*** | | ***C. albicans*** | | ***F. solani*** | | ***A. niger*** | |
|  | **MIC_50_** | **%**  **GI** | **MIC_50_** | **%**  **GI** | **MIC_50_** | **%**  **GI** | **MIC_50_** | **%**  **GI** |
| AHE/F1 | - | -3.52 | - | 1.1 | - | 12.1 | - | -11.6 |
| AHE/F2 | - | -2.51 | - | 1.13 | - | 13 | - | -6.7 |
| AHE/F3 | - | 20.62 | - | 30.3 | 33.9 | 87.2 | 41.5 | 86.3 |
| AHE/F4 | - | 0.29 | - | 4.7 | - | 5.6 | - | 3.2 |
| AHE/F5 | - | 2.92 | - | 2.7 | - | 13.6 | - | 1.7 |
| AHE/F6 | - | 3.35 | - | 1.6 | - | 5.5 | - | 5.2 |
| AHE/F7 | - | 5.89 | - | 8.1 | - | 5.5 | - | 1.1 |
| AHE/F8 | - | 1.88 | - | 1.8 | - | 4.5 | - | 4 |
| AHE/F9 | - | 1.31 | - | 0.1 | - | 6.9 | - | 2.9 |
| AHE/F10 | - | 3.41 | - | 1.5 | - | -26 | - | 6.2 |
| AHE/F11 | - | 1.36 | - | 3.3 | - | 6.6 | - | 1.9 |
| AHE/F12 | - | 3.66 | - | -10.9 | - | 4.6 | - | 2.1 |
| Amphotericin B | - | 94.9 | - | 82.70 | - | 90.1 | - | 92.4 |

-; not done, MIC_50_ (Minimum inhibitory concentration resulting in 50% growth inhibition in µg/ml) and GI (growth inhibition at 200 µg/ml).

Table S5: Antibacterial activity of *A. hydaspica* isolated fractions from AHB by Sephadex LH20 chromatography against *S. aureus*

|  | *Microorganism* | |
| --- | --- | --- |
| Samples | ***S. aureus*** | |
|  | **MIC** | **% GI** |
| AHB/F1 | - | -14 |
| AHB/F2 | - | -10 |
| AHB/F3 | 53.1 | 78.3 |
| AHB/F4 | 17.1 | 90.0 |
| AHB/F5 | 55.2 | 76.6 |
| AHB/F6 | 64.9 | 70.2 |
| AHB/F7 | - | 2 |
| AHB/F8 | - | -3 |
| Tetracyline | - | 96 |

-; not done, MIC_50_ (Minimum inhibitory concentration resulting in 50% growth inhibition in µg/ml) and GI (growth inhibition at 100 µg/ml).

Table S6: Antibacterial activity of *A. hydaspic*a isolated fractions from AHB by Semi-prep RP-HPLC against *S. aureus*

|  | *Microorganism* | |
| --- | --- | --- |
| Samples | ***S. aureus*** | |
|  | **MIC** | **% GI** |
| AHB/F1 | - | -10 |
| AHB/F2 | - | -11 |
| AHB/F3 | - | 10 |
| AHB/F4 | - | 18.3 |
| AHB/F5 | 10.1 | 92.0 |
| AHB/F6 | - | 36.6 |
| AHB/F7 | - | 5.2 |
| AHB/F8 | - | 13.2 |
| AHB/F9 | - | -6 |
| AHB/F10 | - | 1 |
| Tetracyline | - | 96 |

-; not done, MIC_50_ (Minimum inhibitory concentration resulting in 50% growth inhibition in µg/ml) and GI (growth inhibition at 100 µg/ml).

Table S7: ^1^H NMR data of antimicrobial compounds isolated from *A. hydaspica* (Coupling constant J in Hertz)

| Proton | Catechin-3-*O*-gallate  δ in ppm  (C3)b | Methyl gallate  δ in ppm  (C4) c |
| --- | --- | --- |
| H-2 | 4.56 (*d*, J= 7.0Hz) | 7.11 (s) |
| H-3 | 3.97 (m) | 3.79 (s, OCH3) |
| H-4α  Β | 2.84 (*dd*, J = 16.4 Hz, J = 5.3 Hz)  2.5 ( *d*, J=15.8, 8.2 Hz) | - |
| H-6 | 5.86 (*d*, J=2.0 Hz) | 7.11 (s) |
| H-8 | 5.95 (*d*, J=2.0 Hz) | - |
| H- 2' | 6.83 (*d*, J = 1.9 Hz) | - |
| H-5' | 6.76 (*d*, J = 7.9 Hz) | - |
| H-6' | 6.71(*dd*, J = 8.0 Hz, J = 1.5 Hz | - |
| OH-3 | n.r | - |
| Galloyl | 7.045 (s) | - |

Coupling constants (Hz) in parenthesis, a: DMSO-d6, b: Methanol-d4 and c indicates acetone–d6. Dashes indicate that given proton is absent the molecule.

Table S8: ^13^C NMR data of antimicrobial compounds isolated from *A. hydaspica*

| Carbon | Catechin-3-O-gallate  δ in ppm  (CG; C3) b | Methyl gallate  δ in ppm    (MG; C4) c |
| --- | --- | --- |
| C-1 |  | 120.91 |
| C-2 | 81.53 | 108.90 |
| C-3 | 66.96 | 145.12 |
| C-4 | 27.07 | 137.76 |
| C-4a | 105.57 | - |
| C-6 | 100.66 | 108.90 |
| C-8 | 105.57 | - |
| C-5 | 155.38 | 145.12 |
| C-7 | 150.34 | - |
| C-8a | 156.16 | - |
| C-1' | 130.48 | - |
| C-2' | 113.77 | - |
| C-3' | 144.84 | - |
| C-4' | 144.89 | - |
| C-5' | 114.81 | - |
| C-6' | 118.61 | - |
| C-1 galloyl | 119.32 | - |
| C-2 galloyl | 109.18 | - |
| C-3 galloyl | 145.24 | - |
| C-4 galloyl | 139.07 | - |
| C-5 galloyl | - | - |
| C-6 galloyl | - | - |
| COO- | 165.70 | - |
| C=O | - | 166.27 |
| Methyl | - | 51.0 |

a: DMSO-d6, b: Methanol-d4 and c indicates acetone–d6**.** Dashes indicate that given carbon is not present in the molecule.

**Table S9: Docked complexes of bacterial cell surface proteins and methyl gallate and catechin 3-*O*-gallate along with the vina score and cavity size**

| Drug | Protein | Vina score | Cavity size |
| --- | --- | --- | --- |
| Catechin_3-O-gallate | Autolysine (Atl) | -8.8 | 2208 |
|  |  | -8.6 | 1972 |
|  |  | -8.2 | 2389 |
|  |  | -8.1 | 615 |
|  |  | -7.7 | 661 |
|  | Clumping factor A (ClfA) | -9.7 | 1610 |
|  |  | -8 | 193 |
|  |  | -7.8 | 225 |
|  |  | -7.6 | 173 |
|  |  | -7.4 | 227 |
|  | Fibronectin binding adhesin protein FnBPB | -8.3 | 7640 |
|  |  | -7.9 | 202 |
|  |  | -7.3 | 419 |
|  |  | -6.9 | 292 |
|  |  | -6.9 | 215 |
| Methyl gallate | Autolysine (Atl) | -6.2 | 2208 |
|  |  | -5.8 | 2389 |
|  |  | -5.3 | 1972 |
|  |  | -4.8 | 661 |
|  |  | -4.7 | 615 |
|  | Clumping factor A (ClfA) | -6.1 | 1610 |
|  |  | -5.5 | 225 |
|  |  | -5.3 | 193 |
|  |  | -4.8 | 227 |
|  |  | -4.8 | 173 |
|  | Fibronectin binding adhesin protein FnBPB | -5.6 | 7640 |
|  |  | -5.6 | 419 |
|  |  | -5.5 | 215 |
|  |  | -4.8 | 292 |
|  |  | -4.6 | 202 |
